# Supramolecular Synthon Promiscuity in Phosphoric Acid–Dihydrogen Phosphate Ionic Cocrystals

**DOI:** 10.1021/acs.cgd.2c00150

**Published:** 2022-04-19

**Authors:** Molly M. Haskins, Matteo Lusi, Michael J. Zaworotko

**Affiliations:** Department of Chemical Sciences and Bernal Institute, University of Limerick, Limerick V94 T9PX, Ireland

## Abstract

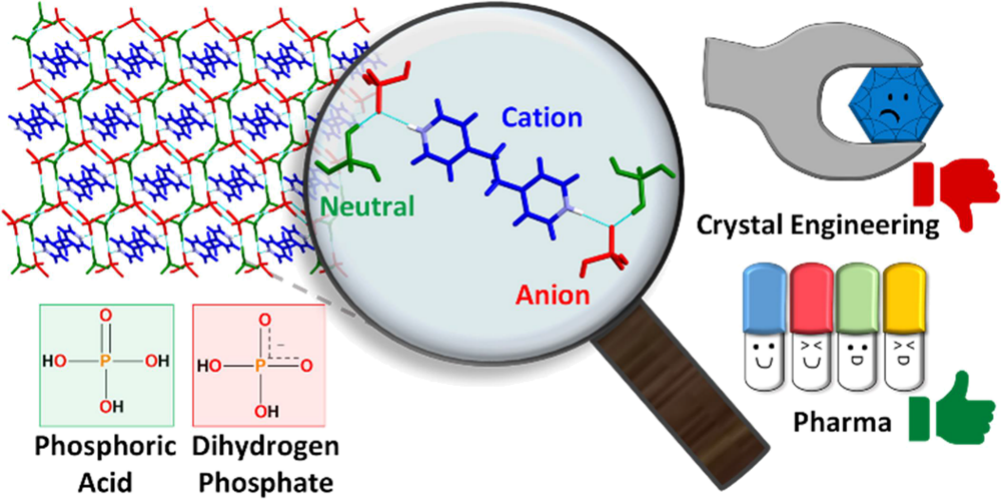

Approximately 80% of active pharmaceutical ingredients (APIs) studied as lead candidates in drug development exhibit low aqueous solubility, which typically results in such APIs being poorly absorbed and exhibiting low bioavailability. Salts of ionizable APIs and, more recently, pharmaceutical cocrystals can address low solubility and other relevant physicochemical properties. Pharmaceutical cocrystals are amenable to design through crystal engineering because supramolecular synthons, especially those sustained by hydrogen bonds, can be anticipated through computational modeling or Cambridge Structural Database (CSD) mining. In this contribution, we report a combined experimental and CSD study on a class of cocrystals that, although present in approved drug substances, remains understudied from a crystal engineering perspective: ionic cocrystals composed of dihydrogen phosphate (DHP) salts and phosphoric acid (PA). Ten novel DHP:PA ionic cocrystals were prepared from nine organic bases (4,4′-bipyridine, 5-aminoquinoline, 4,4′-azopyridine, 1,4-diazabicyclo[2.2.2]octane, piperazine, 1,2-bis(4-pyridyl)ethane, 1,2-bis(4-pyridyl)xylene, 1,2-di(4-pyridyl)-1,2-ethanediol, and isoquinoline-5-carboxylic acid) and one anticonvulsant API, lamotrigine. From the resulting crystal structures and a CSD search of previously reported DHP:PA ionic cocrystals, 46 distinct hydrogen bonding motifs (HBMs) have been identified between DHP anions, PA molecules, and, in some cases, water molecules. Our results indicate that although DHP:PA ionic cocrystals are a challenge from a crystal engineering perspective, they are formed reliably and, given that phosphoric acid is a pharmaceutically acceptable coformer, this makes them relevant to pharmaceutical science.

## Abstract 2

Whereas ionic cocrystals form reliably when using phosphoric acid as a coformer for organic cation dihydrogenphosphate salts, their structures offer a diverse range of hydrogen bonding motifs.

## Introduction

Solid oral dosage forms are a preferred mode of drug administration means that the physicochemical properties of solid forms of drug substances (also known as active pharmaceutical ingredients, APIs) are evaluated at the preclinical stage of drug development.^1−3^ Solid-form screening of APIs, traditionally of salts^4−6^ and polymorphs/solvates,^7−9^ but more recently cocrystals,^10−12^ has for decades been a standard practice in the pharmaceutical industry to identify a solid form(s) suitable for use in a drug product.^13^ Satisfactory aqueous solubility is a primary consideration^14^ during solid form selection as the rate of dissolution, which is controlled by the bulk solubility of the solid form, drives absorption of the API and in turn impacts in vivo performance. Permeability is also a key property that affects absorption of an API, which can therefore be classified by its aqueous solubility and permeability according to the Biopharmaceutical Classification System (BCS).^15^ BCS Class II (low solubility, high permeability) and BCS Class IV (low solubility, low permeability) APIs represent approximately 60% of APIs under development.^16,17^ Polymorphs rarely change aqueous solubility enough to impact in vivo performance,^18^ and multicomponent solid forms such as salts and pharmaceutical cocrystals^19,20^ can significantly impact solubility, sometimes by orders of magnitude.^21^

Cocrystals have been defined as “solids that are crystalline single-phase materials composed of two or more different molecular and/or ionic compounds generally in a stoichiometric ratio, which are neither solvates nor simple salts”.^22^ Pharmaceutical cocrystals, which are typically composed of at least one API and one or more pharmaceutically acceptable coformer,^23^ have grown in interest and not just because they can offer large changes in aqueous solubility. The “cocrystal advantage” comes from several factors: (i) control over physicochemical properties, especially solubility and thermal or hydrolytic stability;^24,25^ (ii) unlike salts, both ionizable and nonionizable molecules are suitable for cocrystal formation;^26−29^ (iii) FDA^30^ and EMA^31^ guidance mean that, unlike salts, bioequivalent cocrystals can be treated as polymorphs, thereby streamlining regulatory approval;^32^ (iv) intellectual property opportunities exist, especially when properties are improved enough for cocrystals to be classified as new drug products.^33^ It seems inevitable that the number of pharmaceutical cocrystals on the market^34^ will grow, driven by the inherent amenability of biologically active molecules to crystal engineering^35−38^ through an understanding of supramolecular synthons.^39^

**Scheme 1:**
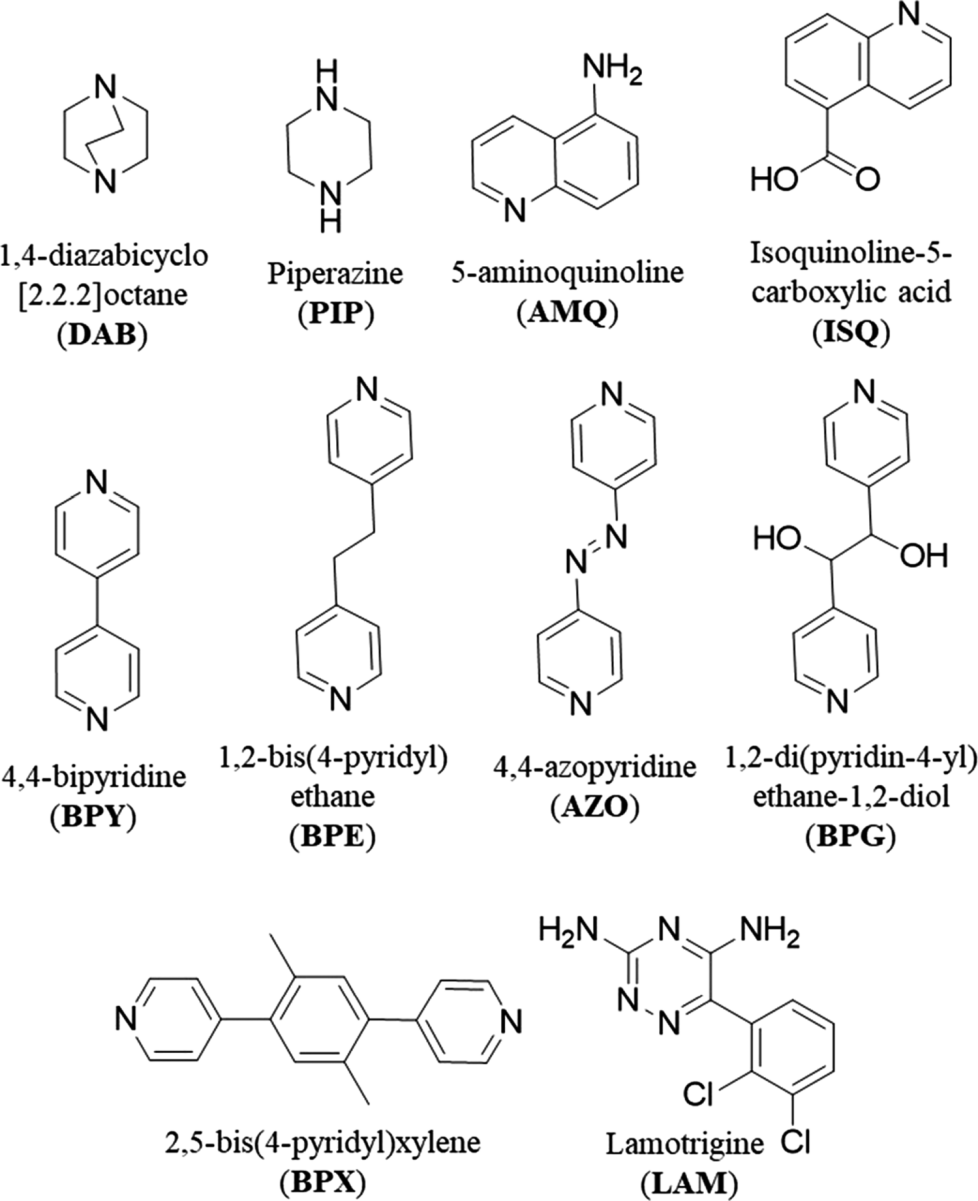
Cocrystal Formers Studied and Their Corresponding Abbreviations These cationic coformers are colored blue herein.

Cocrystals can be classified into two subgroups: molecular cocrystals composed of two or more neutral molecular compounds (coformers); ionic cocrystals (ICCs) formed from a salt and one or more salts or neutral molecular coformers.^40^ ICCs require at least three components, two of which can be varied in a pharmaceutical ICC, affording them a wide diversity of possible compositions and physicochemical properties. Those cocrystals amenable to crystal engineering are driven by the understanding of intermolecular interactions between coformers, typically H-bonded supramolecular synthons,^39^ that typically drive cocrystal formation. However, given that most APIs have multiple H-bonding groups, the hierarchy of supramolecular synthons must be established before families of related cocrystals can be generated by design.^41−46^ A class of ICCs that remains underexplored from a crystal engineering perspective is that formed by dihydrogen phosphate (DHP) anions and phosphoric acid (PA) molecules. DHP anions have long been utilized as counterions for ionizable APIs, as exemplified by Tamiflu, which was reported to offer enhanced pharmacokinetics over its free base.^47^ Furthermore, phosphates are unrestricted for use in drug products as they pose no safety concerns.^4,48^ More recently approved phosphate salts include amifampridine,^49^ which is used for the treatment of Lambert–Eaton myasthenic syndrome. The DHP salt was found to exhibit superior stability compared to its free base and five other salt candidates.^50,51^ Sonidegib phosphate, the API in the basal cell skin cancer drug product Odomzo,^52^ is an ICC comprising DHP and PA molecules. This “diphosphate salt” was developed to overcome solubility concerns and was only later categorized as a DHP:PA cocrystal.^53^ To our knowledge, the first DHP:PA pharmaceutical cocrystal was reported by Chen et al. in 2007. This ionic cocrystal resolved issues relating to the stability of its amorphous free base and salt alternatives.^54^ We report herein a combined experimental (using the coformers presented in Scheme 1) and Cambridge Structural Database (CSD)^55^ mining study of DHP:PA cocrystals to investigate their amenability to crystal engineering for producing ionic pharmaceutical cocrystals.

## Experimental Procedures

Reagents and solvents were obtained from Sigma Aldrich and TCI and used as received.

### Powder X-ray Diffraction (PXRD)

PXRD studies of microcrystalline samples were performed in the Bragg–Brentano geometry on a PANalytical Empyrean diffractometer (40 kV, 40 mA, Cu Kα_1,2_ (λ = 1.5418 Å)). A scan speed of 0.5 s/step (6°/min) with a step size of 0.05° in 2θ was used at ambient temperature.

### Single-Crystal X-ray Diffraction (SCXRD)

Single crystals were manually selected and mounted with paratone oil on a polymeric fiber. Data were collected on a Bruker Quest D8 diffractometer equipped with a Cu-sealed tube (Cu Kα radiation, λ = 1.5418 Å), a Photon II CPAD detector, and an Oxford Cryosystem 800. Data were integrated with the APEX program suite and empirically corrected for absorption correction. Structure solution was found through direct methods in SHELX through XSEED. All heavy atoms were found on the electron density map and refined anisotropically against all F^2^_obb_. Hydrogen atoms were constrained through the riding model in their position as determined by an analysis of the distances between heavy atoms. X-ray crystallographic parameters are tabulated in Table 1.

**Table 1:** Crystallographic Data for the Ten Novel ICCs Prepared Herein

cocrystal	AMQDPP	BPEDPP	PIPDPP	BPYDPP	ISQDPP
molecular formula	C_18_H_25_N_4_O_12_P_3_	C_6_H_12_NO_8_P_2_	C_4_H_22_N_2_O_16_P_4_	C_5_H_10_NO_8_P_2_	C_10_H_13_NO_10_P_2_
**cation**:**DHP:PA**	4:4:2	1:1:1	1:1:1	1:1:1	1:1:1
*M*_r_	582.33	288.11	478.11	274.08	368.155
temp (K)	150 (2)	172.6 (2)	226 (2)	150 (2)	235.2 (2)
crystal system	triclinic	triclinic	monoclinic	monoclinic	orthorhombic
space group	*P*-1	*P*-1	*P*2_1_/*c*	*P*2/*c*	*Pca*2_1_
*a* (Å)	9.8229 (3)	7.856 (6)	7.9712 (2)	8.9320 (2)	12.8691 (3)
*b* (Å)	11.5326 (3)	8.769 (5)	16.1345 (5)	8.9583 (2)	14.8528 (3)
*c* (Å)	22.2937 (7)	8.954 (7)	7.5334 (2)	13.1677 (3)	7.8112 (2)
α (°)	102.0900 (10)	92.49 (3)	90	90	90
β (°)	98.4870 (10)	110.84 (4)	116.3950 (10)	104.5090 (10)	90
γ (°)	103.4980 (10)	104.14 (4)	90	90	90
volume (Å^3^)	2349.46 (12)	553.2 (7)	867.88 (4)	1020.02 (4)	1493.05 (6)
*Z*	4	2	2	4	4
ρ_calc_ g/cm^3^	1.646	1.730	1.830	1.785	1.638
μ (mm^–1^)	2.999	3.945	4.863	4.243	3.183
F(000)	1208.0	298.0	496.0	564.0	761.4
crystal size (mm^3^)	0.200 × 0.200 × 0.100	0.3368 × 0.101 × 0.067	0.100 × 0.100 × 0.050	0.100 × 0.050 × 0.050	0.100 × 0.090 × 0.034
radiation	Cu Kα (λ = 1.54178)	Cu Kα (λ = 1.54178)	Cu Kα (λ = 1.54178)	Cu Kα (λ = 1.54178)	Cu K_α_ (λ = 1.54178)
2Θ range (°)	4.142 to 159.83	10.512 to 134.318	10.966 to 133.214	9.874 to 133.404	5.96 to 133.26
index ranges	–12 ≤ *h* ≤ 12, –12 ≤ *k* ≤ 13, –28 ≤ *l* ≤ 28	–9 ≤ *h* ≤ 9, –8 ≤ *k* ≤ 10, –10 ≤ *l* ≤ 10	–9 ≤ *h* ≤ 9, –19 ≤ *k* ≤ 19, –7 ≤ *l* ≤ 8	–10 ≤ *h* ≤ 10, –10 ≤ *k* ≤ 10, –15 ≤ *l* ≤ 15	–15 ≤ *h* ≤ 15, –14 ≤ *k* ≤ 17, –9 ≤ *l* ≤ 9
reflections collected	41,302	9634	10,346	29,457	14,952
independent reflections	9700 [*R*_int_ = 0.0718, *R*_sigma_ = 0.0559]	1957 [*R*_int_ = 0.0506, *R*_sigma_ = 0.0339]	1525 [*R*_int_ = 0.0483, *R*_sigma_ = 0.0332]	1814 [*R*_int_ = 0.0436, *R*_sigma_ = 0.0175]	2606 [*R*_int_ = 0.0551, *R*_sigma_ = 0.0389]
data/restraints/parameters	9700/0/681	1957/0/196	1525/0/124	1814/0/150	2606/1/214
GOF on F^2^	1.058	1.064	0.985	1.130	1.056
final *R* indexes [*I* > =2σ(*I*)]	*R*_1_ = 0.0815, *wR*_2_ = 0.2235	*R*_1_ = 0.0353, *wR*_2_ = 0.0951	*R*_1_ = 0.0311, *wR*_2_ = 0.0817	*R*_1_ = 0.0329, *wR*_2_ = 0.0790	*R*_1_ = 0.0291, *wR*_2_ = 0.0757
final *R* indexes [all data]	*R*_1_ = 0.0932, *wR*_2_ = 0.2328	*R*_1_ = 0.0360, *wR*_2_ = 0.0959	*R*_1_ = 0.0311, *wR*_2_ = 0.0817	*R*_1_ = 0.0391, *wR*_2_ = 0.0857	*R*_1_ = 0.0293, *wR*_2_ = 0.0759
largest diff. peak/hole/e Å^–3^	0.71/–0.95	0.34/–0.53	0.34/–0.46	0.32/–0.49	0.22/–0.23
CCDC number	2144619	2144618	2144620	2144624	2144622

cocrystal ID	BPGDPP	BPXDPP	LAMDPP	AZODPP	DABDPP
molecular formula	C_12_H_24_N_2_O_18_P_4_	C_18_H_32_N_2_O_18_P_4_	C_18_H_28_Cl_4_N_10_O_17_P_4_	C_10_H_20_N_4_O_16_P_4_	C_6_H_21_N_2_O_12_P_3_
**cation**:**DHP:PA**	1:1:1	2:2:2	2:2:2	1:1:1	2:2:1
*M*_r_	608.21	688.35	922.18	576.18	406.16
temp (K)	173 (2)	150 (2)	100 (2)	150 (2)	153 (2)
crystal system	triclinic	monoclinic	monoclinic	triclinic	monoclinic
space group	P-1	*P*2_1_/*c*	*P*2_1_	*P*-1	*P*2_1_
*a* (Å)	7.2280 (16)	9.0286 (10)	10.3073 (2)	7.9812 (2)	8.705 (2)
*b* (Å)	7.6984 (18)	24.888 (4)	10.2225 (2)	8.6112 (3)	7.820 (2)
*c* (Å)	11.702 (3)	12.7779 (17)	17.3532 (4)	8.7042 (3)	11.573 (2)
α (°)	77.371 (8)	90	90	92.2260 (10)	90.00 (3)
β (°)	80.342 (10)	90.804 (10)	103.4270 (10)	104.8780 (10)	98.11 (3)
γ (°)	65.681 (9)	90	90	108.8310 (10)	90.00 (3)
volume (Å^3^)	576.8 (2)	2871.0 (7)	1778.47 (6)	542.35 (3)	779.9 (3)
*Z*	1	4	2	1	2
ρ_calc_ g/cm^3^	1.751	1.593	1.722	1.764	1.729
μ (mm^–1^)	3.889	3.201	5.512	4.054	4.151
F(000)	314.0	1432.0	940.0	296.0	424.0
crystal size (mm^3^)	0.200 × 0.200 × 0.100	0.200 × 0.100 × 0.100	0.100 × 0.100 × 0.050	0.100 × 0.050 × 0.050	0.100 × 0.100 × 0.050
radiation	CuKα (λ = 1.54178)	CuKα (λ = 1.54178)	CuKα (λ = 1.54178)	CuKα (λ = 1.54178)	CuKα (λ = 1.54178)
2Θ range (°)	12.796 to 133.552	7.104 to 133.676	5.236 to 133.208	10.61 to 133.406	7.716 to 133.428
index ranges	–8 ≤ *h* ≤ 6, –9 ≤ *k* ≤ 9, –13 ≤ *l* ≤ 13	–10 ≤ *h* ≤ 10, –29 ≤ *k* ≤ 29, –15 ≤ *l* ≤ 15	–12 ≤ *h* ≤ 12, –12 ≤ *k* ≤ 12, –20 ≤ *l* ≤ 20	–9 ≤ *h* ≤ 9, –10 ≤ *k* ≤ 10, –10 ≤ *l* ≤ 10	–10 ≤ *h* ≤ 9, –8 ≤ *k* ≤ 9, –13 ≤ *l* ≤ 13
reflections collected	4292	97,317	28,177	12,375	5094
independent reflections	1907 [*R*_int_ = 0.0451, *R*_sigma_ = 0.0532]	5092 [*R*_int_ = 0.1811, *R*_sigma_ = 0.0676]	6285 [*R*_int_ = 0.0862, *R*_sigma_ = 0.0635]	1885 [*R*_int_ = 0.0482, *R*_sigma_ = 0.0319]	2426 [*R*_int_ = 0.0668, *R*_sigma_ = 0.0827]
data/restraints/parameters	1907/0/169	5092/0/407	6285/9/497	1885/0/166	2426/1/215
GOF on F^2^	1.052	1.129	1.035	1.105	1.062
final *R* indexes [*I* > =2σ(*I*)]	*R*_1_ = 0.0498, *wR*_2_ = 0.1412	*R*_1_ = 0.0583, *wR*_2_ = 0.1317	*R*_1_ = 0.0505, *wR*_2_ = 0.1265	*R*_1_ = 0.0440, *wR*_2_ = 0.1279	*R*_1_ = 0.0577, *wR*_2_ = 0.1387
final *R* indexes [all data]	*R*_1_ = 0.0505, *wR*_2_ = 0.1420	*R*_1_ = 0.1286, *wR*_2_ = 0.1851	*R*_1_ = 0.0622, *wR*_2_ = 0.1369	*R*_1_ = 0.0448, *wR*_2_ = 0.1289	*R*_1_ = 0.0693, *wR*_2_ = 0.1509
largest diff. peak/hole/e Å^–3^	0.63/–0.45	0.47/–0.75	0.97/–0.50	0.38/–0.57	0.59/–0.59
CCDC number	2144615	2144623	2144621	2144617	2144616

### CSD Analysis

A CSD search was conducted using v5.43 (Nov 2021 update) to identify structures that contain DHP anions and PA molecules. The following restrictions were applied: 3D coordinates; organics; single crystal structures only. The resulting hits were manually filtered to identify ionic cocrystal entries. The following parameters were addressed after filtering: (i) number of structures with both PA molecules and DHP anions; (ii) number of ionic DHP cocrystals that do not have PA as their neutral component; (iii) average P–O bond distances for P–O and P–OH; (iv) hydrogen bond motifs (HBM) formed between DHP and PA; and (v) (O···O) between DHP and PA.

### Cocrystals

The cocrystal formers used to prepare cocrystals 1–10 are illustrated in Scheme 1. Experimental details of each of the solution crystallizations are presented below.

#### Cocrystal 1: [4,4’-Bipyridine]-1,1′-diium Dihydrogen Phosphate-Phosphoric Acid (BPYDPP)

4,4′-Bipyridine (15.6 mg, 0.1 mmol) and crystalline PA (39.2 mg, 0.4 mmol) were dissolved in 1:1 ethanol:water (3 mL). The solvent slowly evaporated to afford colorless rod crystals.

#### Cocrystal 2: 4,4′-(Ethane-1,2-diyl)bis(pyridin-1-ium) Dihydrogen Phosphate-Phosphoric Acid (BPEDPP)

1,2-Bis(4-pyridyl)ethane (18.4 mg, 0.1 mmol) and crystalline PA (39.2 mg, 0.4 mmol) were dissolved in 1:1 ethanol:water (3 mL). The solvent slowly evaporated to form a clear oil. This vial was transferred into a larger vial for vapor diffusion with acetonitrile to produce colorless plates.

#### Cocrystal 3:[4,4′-Azopyridine]-1,1′-diium Dihydrogen Phosphate- Phosphoric Acid (AZODPP)

4,4′-Azopyridine (18.4 mg, 0.1 mmol) and crystalline PA (39.2 mg, 0.4 mmol) were dissolved in 1:1 methanol:water (3 mL). The solvent slowly evaporated to generate dark red needle crystals.

#### Cocrystal 4: 4,4′-(2,5-Dimethyl-1,4-phenylene)bis(pyridin-1-ium) Dihydrogen Phosphate- Phosphoric Acid (BYXDPP)

2,5-bis(4-pyridyl)xylene (26.0 mg, 0.1 mmol) and crystalline PA (39.2 mg, 0.4 mmol) were dissolved in 1:1 ethanol:water (3 mL). Slow solvent evaporation afforded colorless rod crystals.

#### Cocrystal 5: 4,4′-(1,2-Dihydroxyethane-1,2-diyl)bis(pyridin-1-ium) Dihydrogen Phosphate (BPGDPP)

1,2-di(pyridin-4-yl)ethane-1,2-diol (21.6 mg, 0.1 mmol) and crystalline PA (39.2 mg, 0.4 mmol) were dissolved in 1:1 ethanol:water (3 mL). The solvent slowly evaporated, forming a clear oil. This vial was transferred into a larger vial for vapor diffusion with acetonitrile to afford block crystals.

#### Cocrystal 6: Piperazine-1,4-diium Dihydrogen Phosphate–Phosphoric Acid (PIPDPP)

Piperazine (8.6 mg, 0.1 mmol) and crystalline PA (39.2 mg, 0.4 mmol) were dissolved in deionized water (1 mL). This vial was transferred into a larger vial for vapor diffusion with acetonitrile to produce colorless block crystals.

#### Cocrystal 7: 5-Aminoquinolin-1-ium Dihydrogen Phosphate–Phosphoric Acid (AMQDPP)

5-aminoquinoline (14.4 mg, 0.1 mmol) and crystalline PA (19.6 mg, 0.2 mmol) were dissolved in 2:1 methanol:water (4 mL). The solvent slowly evaporated to afford dark red–brown needle crystals.

#### Cocrystal 8: 1,4-Diazabicyclo[2.2.2]octane-1,4-diium Dihydrogen Phosphate–Phosphoric Acid (DABDPP)

1,4-Diazabicyclo[2.2.2]octane (DABCO) (22.4 mg, 0.2 mmol) and crystalline PA (76.8 mg, 0.8 mmol) were dissolved in deionized water (2 mL). This vial was transferred into a larger vial for vapor diffusion with acetonitrile to afford colorless needle crystals.

#### Cocrystal 9: 5-Carboxyisoquinolin-2-ium Dihydrogen Phosphate–Phosphoric Acid (ISQDPP)

Isoquinoline-5-carboxylic acid (17 mg, 0.1 mmol) and crystalline PA (19.6 mg, 0.2 mmol) were dissolved in 2:1 methanol:water (2 mL). The solvent slowly evaporated to afford light yellow rod crystals.

#### Cocrystal 10: Lamotrigine Dihydrogen Phosphate–Phosphoric Acid (LAMDPP)

Lamotrigine (25.6 mg, 0.1 mmol) and crystalline PA (19.6 mg, 0.2 mmol) were dissolved in 2:1 ethanol:water (3 mL). The solvent slowly evaporated to afford colorless plate crystals.

## Results and Discussion

### CSD Analysis of DHP:PA Cocrystals

Our CSD survey revealed 557 entries for DHP in organic structures, 64 of which can be identified as ICCs that do not contain PA as their neutral component (Table S2.1). Interestingly, 53 of the 64 structures were found to contain both tetrabutylammonium and DHP with nitrogen bases such as pyridines or amines. These structures were originally targeted in the context of anion binding (allosteric regulation) and anion receptor recognition studies in protic solvents.^56−58^

A second CSD search revealed 140 entries comprising at least one neutral PA, of which 55 were found to also contain DHP (Table S2.2). Among these 55 entries, only six structures were originally identified as cocrystals,^54,59−62^ but almost 40% of the entries were published prior to the term “*ionic cocrystal*” being coined in 2010.^40^ Rather, terms such as H_2_PO_4_^–^-H_3_PO_4_ clusters,^63^ adducts,^64^ or PA solvates^65^ were used. In general, these DHP:PA ICCs were isolated serendipitously whilst targeting DHP salts. Three DHP:PA pharmaceutical cocrystals were found in this library of ICCs: the first DHP:PA pharmaceutical cocrystal reported by Chen et al. in 2007 (REFCODE: PETTIS);^54^ aripiprazole, which is used in the treatment of schizophrenia and bipolar disorder (REFCODE: ELEVOI) and^60^ orbifloxican, an antimicrobial veterinary drug used for the treatment of gastrointestinal and respiratory infections (CURJAD).^61^

### Bond Distances and Their Utility To Distinguish between DHP and PA

**Scheme 2:**
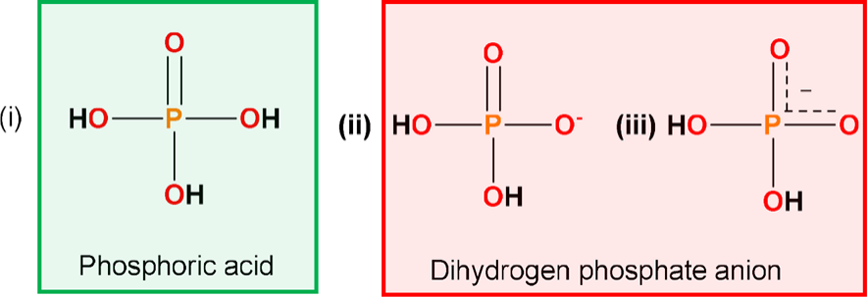
Structures of Phosphoric Acid (PA) Are Colored Green (i) and Dihydrogen Phosphate (DHP); (ii) and (iii) are Colored Red Herein

Information regarding the composition and ionization state of molecular crystals can be derived from the analysis of their crystal structures, typically obtained by SCXRD. However, SCXRD has limitations with respect to the location of protons,^66^ a critical matter with respect to differentiating between DHP anions and PA molecules. PA is composed of one P=O and three P–OH moieties. When PA is deprotonated, the electron density is delocalized over two P–O bonds (Scheme 2(iii)). Mogul was initially used to determine the average bond lengths of P–OH and P–O but inconsistent labeling for DHP (Scheme 2(ii) and (iii)) prompted us to use Conquest (v5.43, Nov 2021 update). Searches on structures (i) and (ii) in Scheme 2 revealed an average P=O distance of 1.5056 ± 0.01396 Å for (i) and an average P–O^–^ distance of 1.5095 ± 0.01595 Å for (ii). Therefore, P–O^–^ and P=O bonds cannot be differentiated and are classified as “P–O bonds” herein (see Section S2.3 for more information).

**Chart 1:**
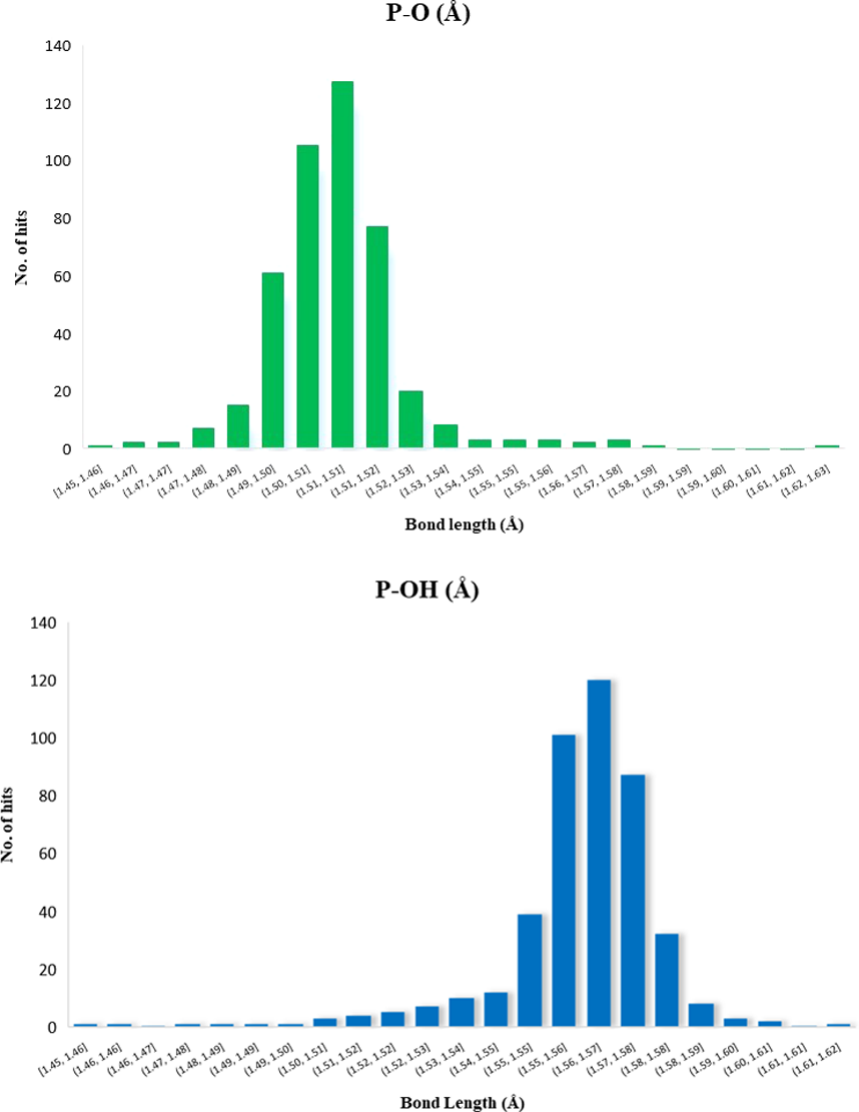
Histograms of P–O (Å) (top) and P–OH (Å) (Bottom) Bond Lengths in Crystal Structures Deposited in the CSD

P–OH and P–O bond lengths were, however, found to be statistically different. As detailed in Scheme 2(iii), P–O distances average 1.5079 ± 0.0148 Å, whereas P–OH distances average 1.5603 ± 0.0166 Å (Chart 1). P–OH and P–O bond lengths are therefore used herein to distinguish between DHP anions and PA molecules. A caveat is that the data for the structures in our library were collected at different temperatures and a study by Voguri et al. on agomelatine-phosphate revealed that proton migration (transformation from salt to cocrystal) occurred upon heating to 330 K.^67^ Nevertheless, all structures were in good agreement with average P–O and P–OH distances.

O···O contacts between PA and DHP were also evaluated to determine if they might aid in distinguishing between DHP and PA as we anticipated that charge-assisted H-bonding might be distinctive (see Section S2.4). No significant differences in O···O distances were observed (average 2.546 Å, Figure S2.1, indicative of strong H-bonding^68,69^).

### Cocrystals 1–10

The following *N*-heterocyclic organic bases were selected for study based upon having p*K*_a_ values from 4 to 8: 4,4′-bipyridine, 5-aminoquinoline, 4,4′-azopyridine, DABCO, piperazine, 1,2-bis(4-pyridyl)ethane, 1,2-bis(4-pyridyl)xylene, 1,2-di(4-pyridyl)-1,2-ethanediol, isoquinoline-5-carboxylic acid, and one anticonvulsant API, lamotrigine (Scheme 1). Each base was cocrystallized with PA to afford single crystals suitable for SCXRD analysis (see Section S1 of the Supporting Information for more details).

**Figure 1:**
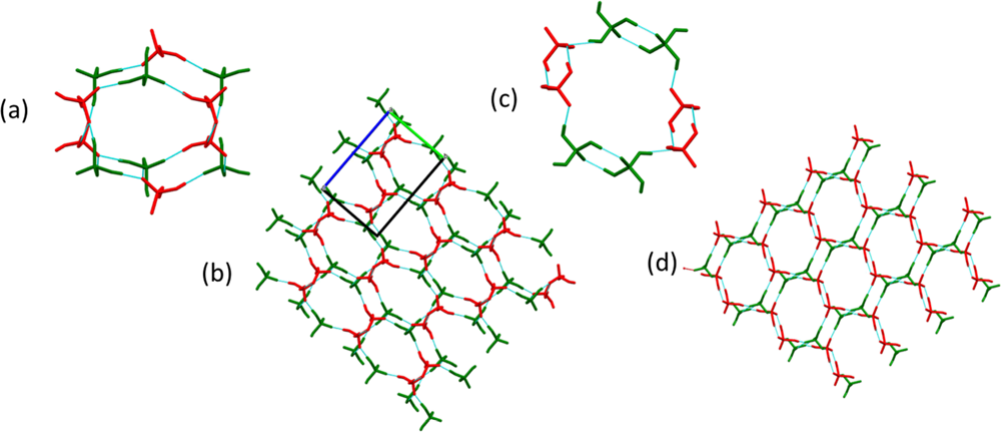
Hydrogen-bonded motif in cocrystal **1**, (a) 3D network in **1**, (b) hydrogen-bonded motif in cocrystals **2** and **3** (c) and the 3D network in **2** and **3**, (d).

**Scheme 3:**
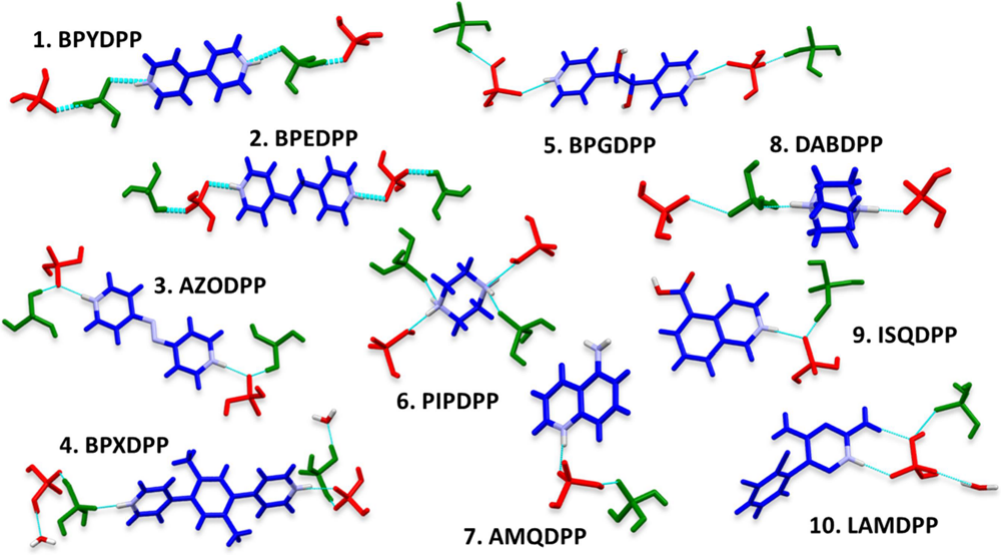
Local Environments of the Cations (blue), DHP Anions (red), PA Molecules (green), and Water Molecules in the novel ICCs Reported Herein

The new DHP:PA ionic cocrystals can be grouped based on common structural features and the local arrangement of cations, anions and molecules for each structure is presented in Scheme 3. Cocrystals **1**–**4** and **8** form channel structures in which DHP anions and PA molecules form a network and organic cations reside in cavities. BPYDPP (**1**) crystallized in *P*2/*c* with one DHP anion, one PA molecule and 0.5 BPY cations in the asymmetric unit. The BPY pyridinium moiety interacts with PA anions (N1···O4A, 2.771 Å). PA molecules and DHP anions form a 3D H-bonded network composed of alternating DHP and PA tetramers (Figure 1a) that afford cavities (Figure 1b) along the *a-*axis in which BPY cations reside (Figure S1.1). BPEDPP (**2**) and AZODPP (**3**) both crystallized in *P*-1 with one DHP anion, one PA molecule, and 0.5 cations in their asymmetric units. The pyridinium moieties of BPE and AZO form H-bonds to a DHP oxygen (BPEDPP, N1···O4, 2.801 Å; AZODPP, N1···O3, 2.728 Å). DHP anions form dimers that create an eight-membered ring motif composed of four alternating DHP and PA dimers (Figure 1c). **2** and **3** form 3D networks (Figure 1d) with channels along the *b-*axis in **2**, with BPE forming no close contacts with other BPEs (Figure S1.2), and channels along the *c*-axis in **3** (Figure S1.3), with azo moieties weakly interacting with DHP anions (N2···O4, 3.033 Å) and π–π stacking (4.155 Å) with adjacent AZO cations.

BPXDPP (**4**) crystallized in *P*2_1_/*c*. Its asymmetric unit contains one BPX cation, two DHP anions, two PA molecules, and two water molecules. One end of BPX H-bonds with DHP (N3···O2B, 2.666 Å), the other end with PA (N16···O3D, 2.682 Å). BPX cations form a herringbone motif surrounded by DHP:PA chains that are bridged by water molecules along the *c*-axis to afford a 3D network (Figure S1.4). The BPX cations in the channels exhibit π–π interactions with adjacent BPX cations (4.513, 4.854, and 4.513 Å for the three rings). DABDPP (**8**) crystallized in *P*2_1_ and is composed of one DAB cation, two DHP anions, and one PA molecule in its asymmetric unit. Like **4**, DAB forms charge-assisted H-bonds with both DHP anions (N1···O21A, 2.576 Å) and PA molecules (N2···O4, 2.734 Å). DHP anions and PA molecules form a 3D network with DAB cations sitting in cavities along the *a*-axis (Figure S1.8).

**Figure 2:**
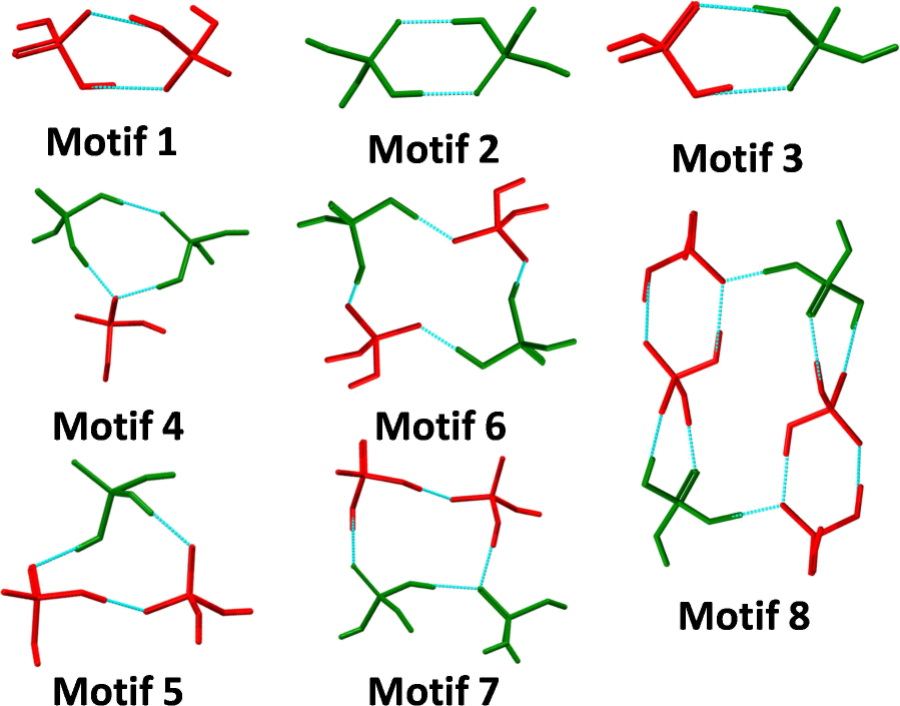
Eight most common HBMs formed between DHP anions and PA molecules.

Cocrystals **5**, **7**, **9,** and **10** all exhibit layered structures composed of H-bonded sheets or chains of DHP anions and PA molecules. The resulting sheets are layered in various ways that can be defined by the role of the cations. BPGDPP (**5**) crystallized in *P*-1 with one DHP anion, one PA molecule, and 0.5 BPG cations in the asymmetric unit. DHP anions interact with the pyridinium moiety of BPG (N14···O5A, 2.686 Å) and hydroxyl moieties (O8···O2A, 2.703 Å). DHP anions and PA anions form a tetramer (Figure 2, motif *6*) that propagates into sheets along the *a* and *b* axes. BPG cations pack parallel to the *a*-axis to form a layered structure (Figure S1.5). **5** did not exhibit a 3D H-bonded network. AMQDPP (**7**) crystallized in *P*-1 with four AMQ cations, four DHP anions, and two PA molecules in the asymmetric unit. All four independent pyridinium rings interact with a DHP anion (N1A···O4C, 2.810 Å, N1B···O1A, 2.728 Å, N1C···O3E, 2.693 Å, N1D···O2B, 2.874 Å) while two of the amino groups H-bond with two PA molecules (N11B···O1F, 2.904 Å, N11D···O4D, 2.986 Å) and the remaining two with DHP anions (N11A···O4E, 2.935 Å) with one of the amino groups simultaneously H-bonding with two DHPs (N11C···O4A, 2.925 Å, N11C···O2E, 3.009 Å). AMQ cations form a π stacked pillar oriented along the crystallographic 1,1,0 direction. DHP and PA moieties stack along the *a* axis, and these chains H-bond to form sheets. The AMQ pillars and DHP:PA sheets alternate to generate a layered structure that stacks along the *c* axis. (Figure S1.7).

ISQDPP (**9**) also forms a layered structure and crystallized in *Pca*2_1_ with one DHP anion, one PA molecule, and one ISQ cation in the asymmetric unit. DHP anions interact with pyridinium rings (N1···O4, 2.267 Å) while the carboxylic acid forms H-bonds with PA molecules (O9···O7, 2.647 Å). DHP:PA H-bond to form a ribbon along the *a*-axis. These ribbons stack along the *c* axis to form a sheet that is parallel to the *ac* plane and surrounds inversely packed ISQ cations (Figure S1.9). LAMDPP (**10**) is a pharmaceutical ICC comprising the anticonvulsant drug lamotrigine (LAM). **10** is an isolated site hydrate that crystallized in *P*2_1_ with two LAM cations, two DHP anions, two PA molecules, and one water molecule in the asymmetric unit. The LAM cations stack with the nonprotonated pyridyl moieties of one LAM cation H-bonding to the amino group of the other (N14A···N4B, 3.093 Å) while the halogenated rings face out in opposite directions in the perpendicular plane to the pyridyl rings. LAM pairs are stacked in a staggered fashion perpendicular to each other along the *a* and *b* axes. The protonated nitrogen atom and the adjacent amino group on both LAMs form dimers, one with DHP anions (N2B···O4C, 2.763 Å, N13D···O3C, 2.847 Å), the other with PA molecules (N13A···O1F, 2.896 Å, N2A···O4F, 2.755 Å). DHP:PA ribbons propagate along the *b* axis and are bridged by water molecules (Figure S1.10).

PIPDPP (**6**) crystallized in *P*2_1_*/c* with one DHP anion, one PA molecule, and 0.5 PIP cations in its asymmetric unit. 6 is an outlier in the ICCs reported herein as the tertiary NH_2_^+^ moiety simultaneously H-bonds to DHP and PA. PIP is surrounded by eight alternating DHP and PA molecules and four (two DHP anions and two PA molecules) stacked above and below (Figure S1.6).

The CNC angle (**<_CNC_**) of the cations and the P–O distances of the ten novel ICCs are presented in Table 2. The **<_CNC_** was used to assess the protonation state of the organic bases, whereas P–O distances were used to distinguish DHP anions vs PA molecules. Eight of the ten structures reported herein contain a pyridinium ring. All eight structures have **<_CNC_** greater than 120°, which is indicative of protonation.^71^ This follows the Δp*K*_a_ rule^70^ as all Δp*K*_a_ values of the organic molecules in this study and PA fall between 0.82 and 7.67 (Table S2.4).

**Table 2:**
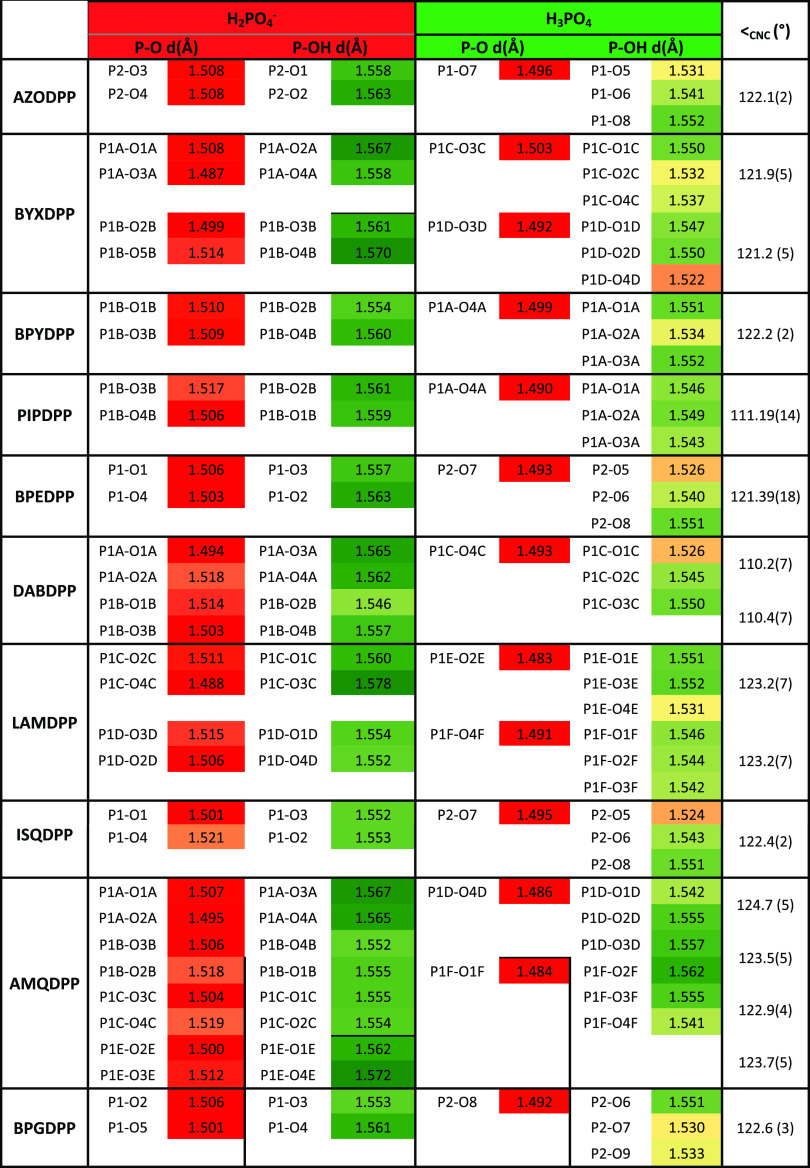
Summary of P–O Bond Distances in the Ten Novel Cocrystal Reported Herein^a^

a Bond lengths equal to or less than 1.507 Å are colored red (delocalized PO moieties in DHP or P=O moiety of PA). Bond lengths equal to or greater than 1.5603 Å are colored green (P–OH moieties). Intermediate distances are highlighted in yellow.

With respect to P–O distances, bond lengths <1.507 Å are colored red and classified as delocalized PO moieties in DHP or P=O moieties in PA. Bond lengths >1.560 Å are colored green and correspond to P–OH moieties. Intermediate distances are given in yellow. Table 2 reveals that the P–O and P–OH distances of all ten ICCs studied herein are consistent with expected values.

With respect to the arrangement of cations, DHP anions and PA molecules, in three of the structure cations, interact with PA molecules, which is contrary to the generally accepted rule that the strongest H-bond donor will engage the strongest H-bond accepter.^72^ Nevertheless, all H-bonds formed by cations herein can be classified as charge-assisted H-bonds.

### Hydrogen Bond Motifs

Initially, the HBMs of the 55 DHP:PA ICCs deposited in the CSD were analyzed to determine if any preferred motifs exist that could be amenable to crystal engineering studies.^73^ For clarity, we distinguish a *supramolecular synthon* as a supramolecular interaction with a characteristic geometry between functional groups^39^ and a *motif* as a network composed of one type of hydrogen-bonded synthon.^74^ Among the 55 ICCs archived in the CSD, eleven were excluded because of omitted hydrogen atoms or disorders (Table S2.2). The remaining 44 revealed, perhaps remarkably, 39 distinct HBMs containing DHP anions, PA molecules and, in nine instances, water molecules (i.e., hydrates). The HBMs of the 44 structures are given in Table S3.1. Figure 2 presents the eight most common HBMs in this library with DHP anions and PA molecules colored red and green, respectively.

Motifs *1*–*3* are DHP-DHP, PA-PA, and DHP-PA dimers, respectively. Motif *1* is the most common HBM, occurring in 25 out of the 44 structures. Five different types of trimer motifs were identified, the most common being composed of two PA molecules and one DHP anion (motif *4*), which was found in 8 structures, and two DHP anions + one PA molecule (motif *5*), present in 14 entries. Twelve distinct tetramer motifs were observed, the most common comprising two DHP anions and two PA molecules, motifs *6* and *7*, which occurred in 11 and 14 structures, respectively. Motif *8*, the next most frequently observed HBM is a ring composed of four DHP anions and two PA molecules, which appeared in 5 entries. The occurrence of these motifs is given in Chart S3.1.

The HBMs in the ten novel ICCs reported herein afforded an additional seven motifs, meaning that 46 distinct HBMs have thus far been observed in 54 DHP:PA ICC crystal structures. The largest HBM is a ring comprising four sets of alternating DHP-DHP and PA-PA dimers and was identified in **2** and **3** (Figure 1c). The most prevalent HBM is a tetramer, exhibited by all ICCs herein except **10**. **4** and **9** exhibit motif *7*, whereas the other seven structures exhibit motif *6*. Motif *6* is the only HBM present in **5**. Indeed, **5** is the only ICC in this study and the CSD that exhibits a single motif. Most structures exhibit three or four HBMs, while two hydrated ICCs exhibited seven different HBMs (Refcodes: IPIPED and HEGDED).

## Conclusions

This study reports the synthesis and single crystal structures of ten novel DHP:PA ICCs comprising protonated *N*-heterocyclic bases, DHP anions, and PA molecules, including an ICC of the BCS class II anticonvulsant lamotrigine. Phosphates are known to form strong H-bonds that generate supramolecular networks of varying dimensionality such as ribbons,^75^ chains,^76^ and layers.^77^ A high degree of promiscuity is also evident in the new ICCs reported herein and those ICCs archived in the CSD. Thirty-nine distinct HBMs were observed in the 44 ordered DHP:PA ICCs archived in the CSD and additional seven motifs were found in the ten novel ICCs reported herein. The diversity of structures formed by DHP:PA ICCs is a challenge to crystal engineers and, like hydrates,^78^ they can be regarded as being a nemesis of crystal engineering and an interesting challenge to crystal structure prediction. Nevertheless, ICCs were found to form reliably, and we consider such ICCs to be relevant to pharmaceuticals for improving the physicochemical properties of ionizable APIs as phosphoric acid is inexpensive, has a low molecular weight, and is FDA-approved. In addition, from a material property perspective, such ICCs may exhibit superior dielectric properties vs the corresponding DPA salts.^79^

## Supporting Information Available

Crystallographic figures for new crystal structures, CSD search parameters and results, and hydrogen bond motifs (PDF)

## Accession Codes

CCDC 2144615–2144624 contain the supplementary crystallographic data for this paper. These data can be obtained free of charge via www.ccdc.cam.ac.uk/data_request/cif, or by emailing data_request@ccdc.cam.ac.uk, or by contacting The Cambridge Crystallographic Data Centre, 12 Union Road, Cambridge CB2 1EZ, UK; fax: +44 1223 336033.
