# Supplementary material for: Supramolecular Synthon Promiscuity in Phosphoric Acid–Dihydrogen Phosphate Ionic Cocrystals
Source: Cryst Growth Des. 2022 Apr 19;22(5):3333–42. doi: 10.1021/acs.cgd.2c00150 (PMC9073934; doi:10.1021/acs.cgd.2c00150)
Supplement: Supplementary file 1 — cg2c00150_si_001.pdf [file cg2c00150_si_001.pdf]

Supporting Information

Supramolecular Synthon Promiscuity in Phosphoric Acid-Dihydrogen Phosphate Ionic Cocrystals

Molly M. Haskins, Matteo Lusi and Michael J. Zaworotko*

*Department of Chemical Sciences, Bernal Institute, University of Limerick, Limerick, Republic of Ireland

Section S1: New crystal structures. 1
1.1. BPYDPP 2
1.2. BPEDPP....................3
1.3. AZODPP....................4
1.4. BPXDPP 5
1.5. BPGDPP 6
1.6. PIPDPP 7
1.7. AMQDPP....................8
1.8. DABDPP....................10
1.9. ISQDPP....................11
1.10. LAMDPP 12
Section S2: CSD Survey 13
Section 2.1. Table of DHP ICCs that do not contain PA as the neutral component found on the CSD 13
Section 2.2. Table of DHP:PA cocrystals....................14
Section 2.3. Bond lengths of P-O and P=O (Å)....................15
S2.4. (O···O) bond distance between DPA and PA (Å)....................16
S2.5. ΔpKa Rule 18
Section S3: Hydrogen bond motifs (HBMs)....................19

Section S1: New crystal structures.

The diagrams below show cations in blue, dihydrogen phosphate (DPA) anions in red and phosphoric acid (PA) molecules in green.

1.1. BPYDPP

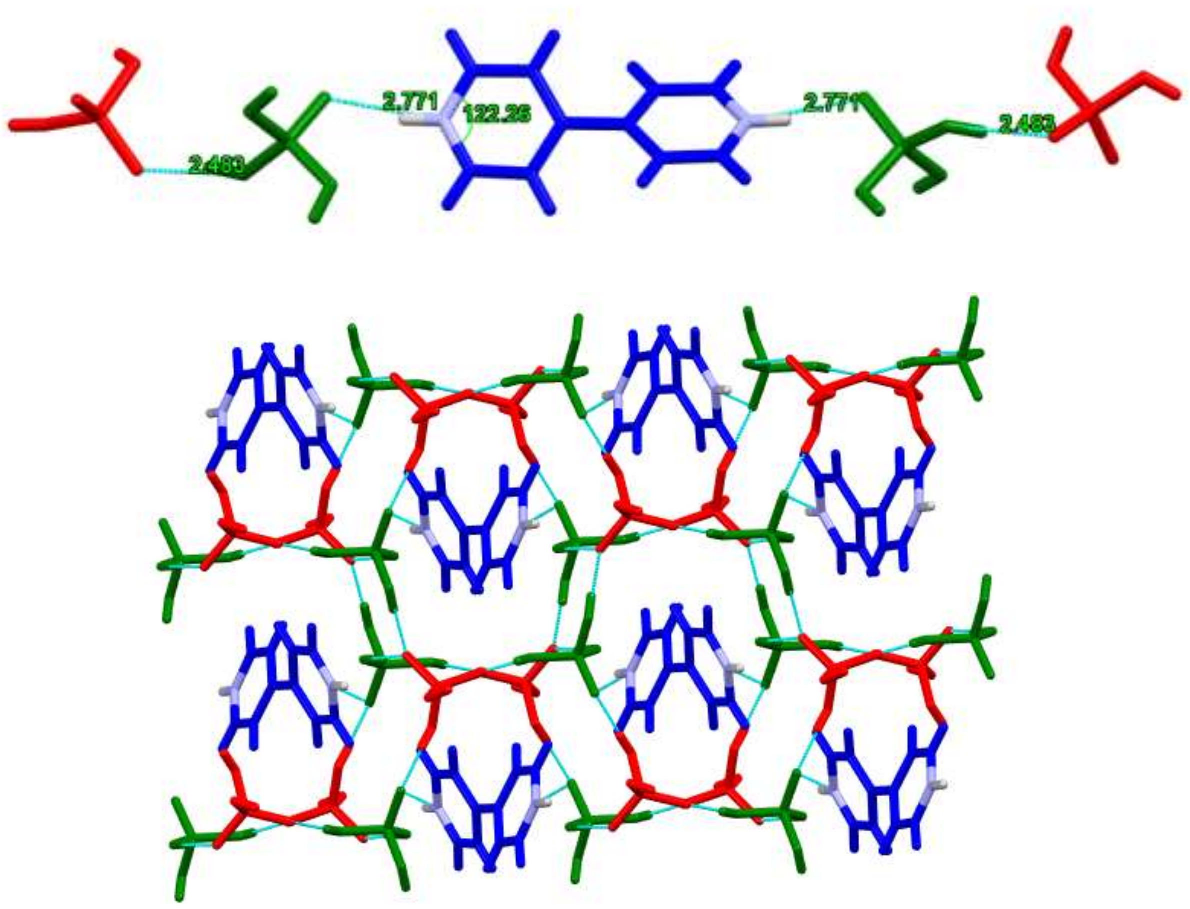

Figure S1.1. *(Top) Structure of BPYDPP, bond lengths in Å and angle in degrees (°) (bottom) packing of BPYDPP along a-axis.*

1.2. BPEDPP

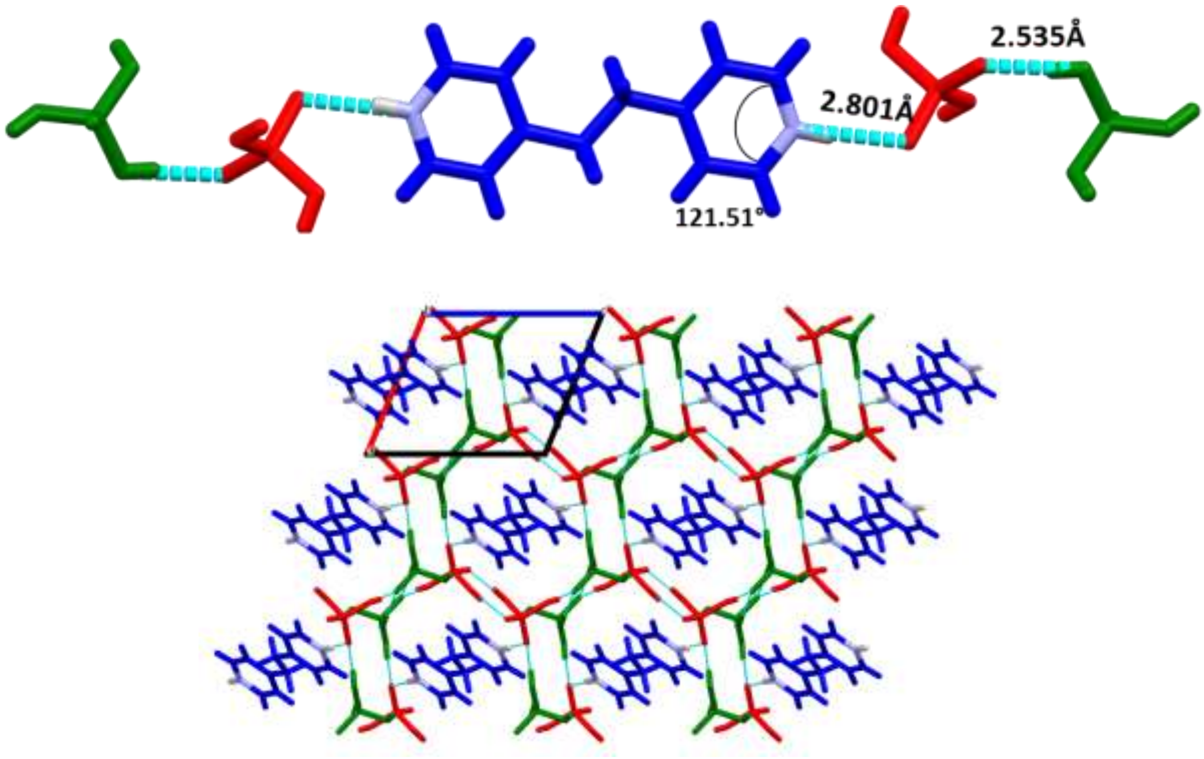

Figure 1.2. *(Top) Structure of BPEDPP, bond lengths in Å and angle in degrees (°) (bottom) packing of BPEDPP along b-axis*

1.3. AZODPP

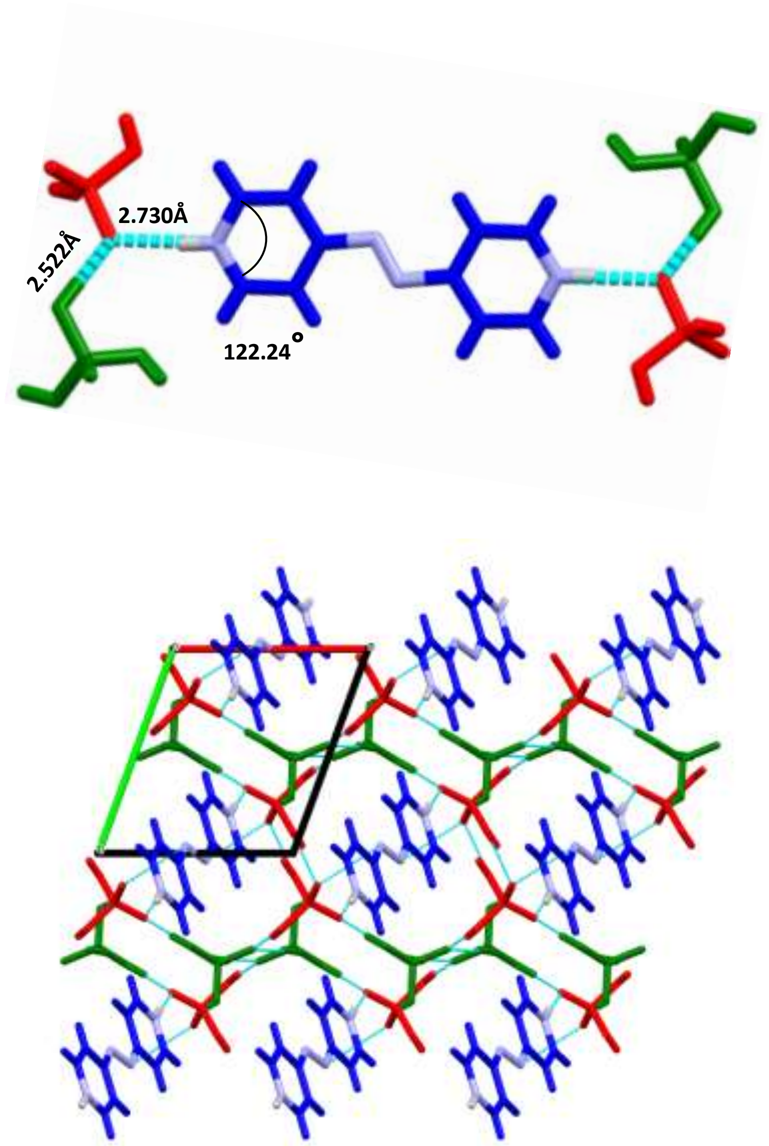

Figure S1.3. *(Top) Structure of AZODPP, bond lengths in Å and angle in degrees (°) (bottom) packing of AZODPP along c-axis*

1.4. BPXDPP

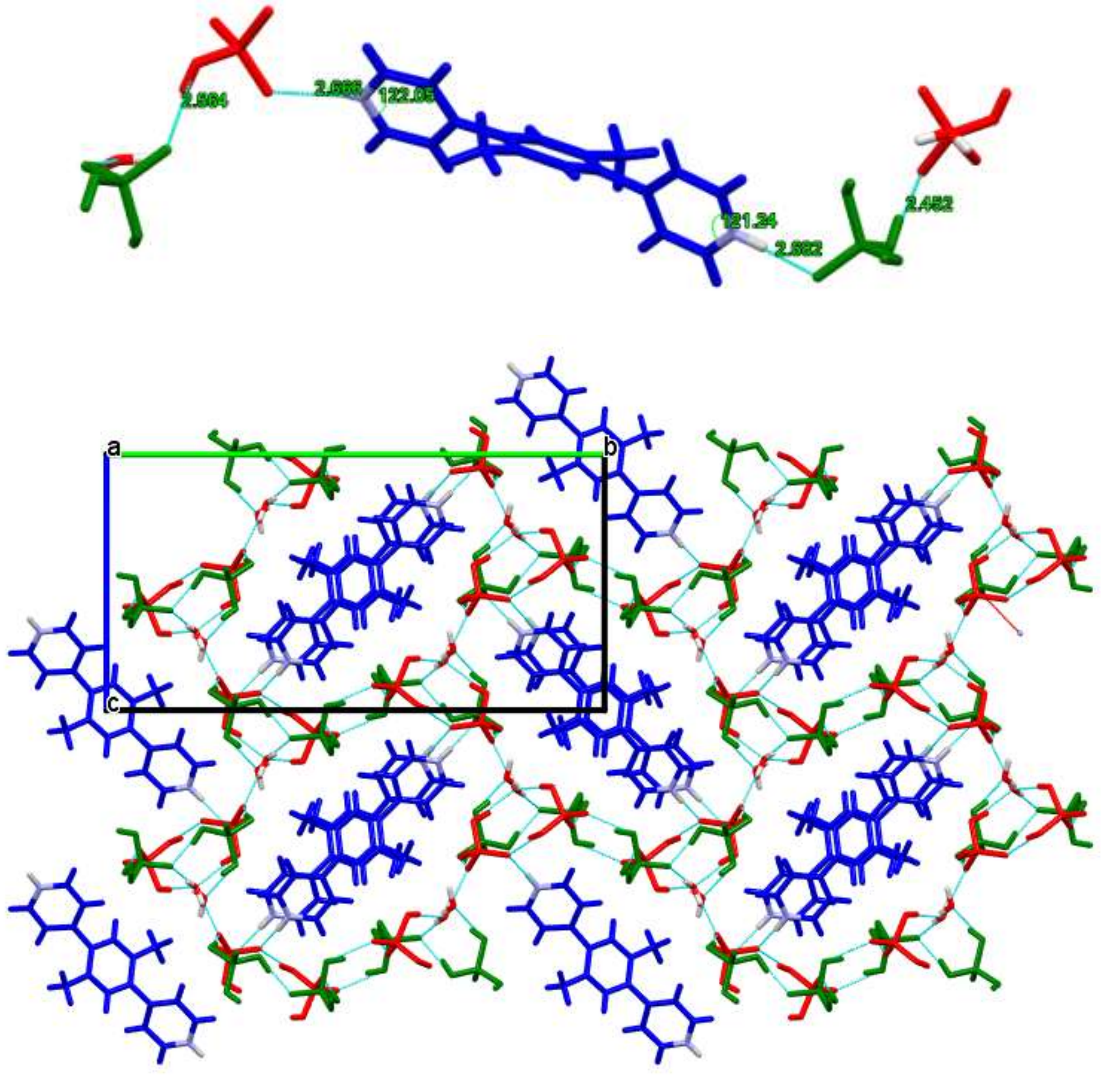

Figure S1.4. *(Top) Structure of BPXDPP, bond lengths in Å and angle in degrees (°) (bottom) packing of BPXDPP along a-axis*

1.5. BPGDPP

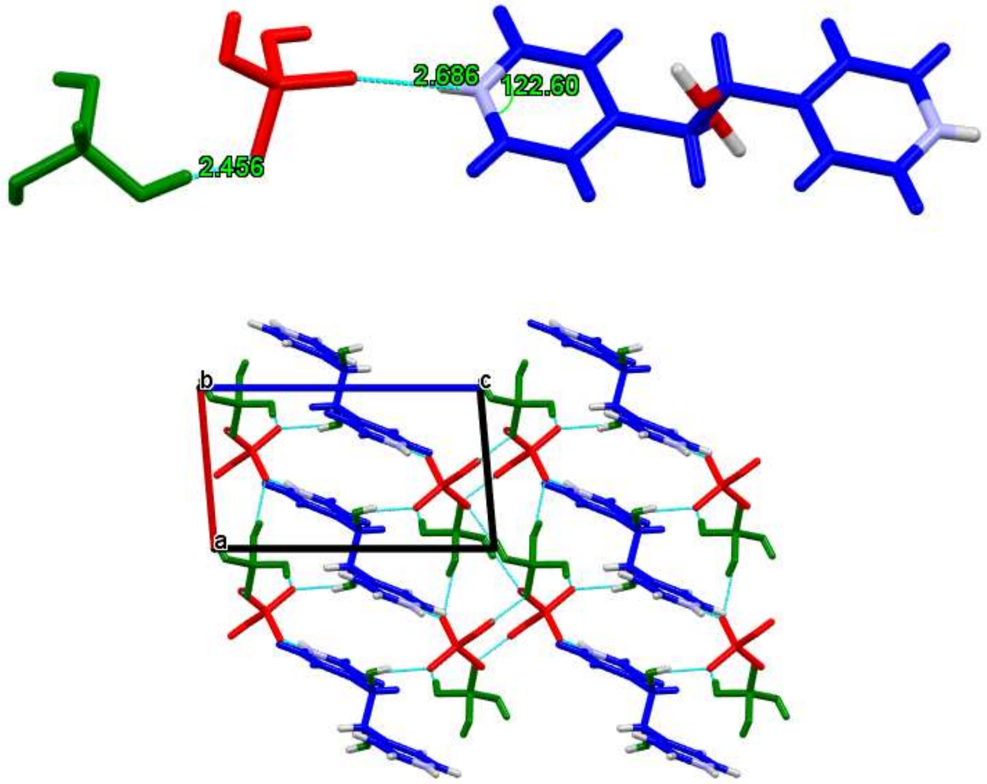

Figure S1.5. *(Top) Structure of BPGDPP, bond lengths in Å and angle in degrees (°) (bottom) packing of BPGDPP along b-axis.*

1.6. PIPDPP

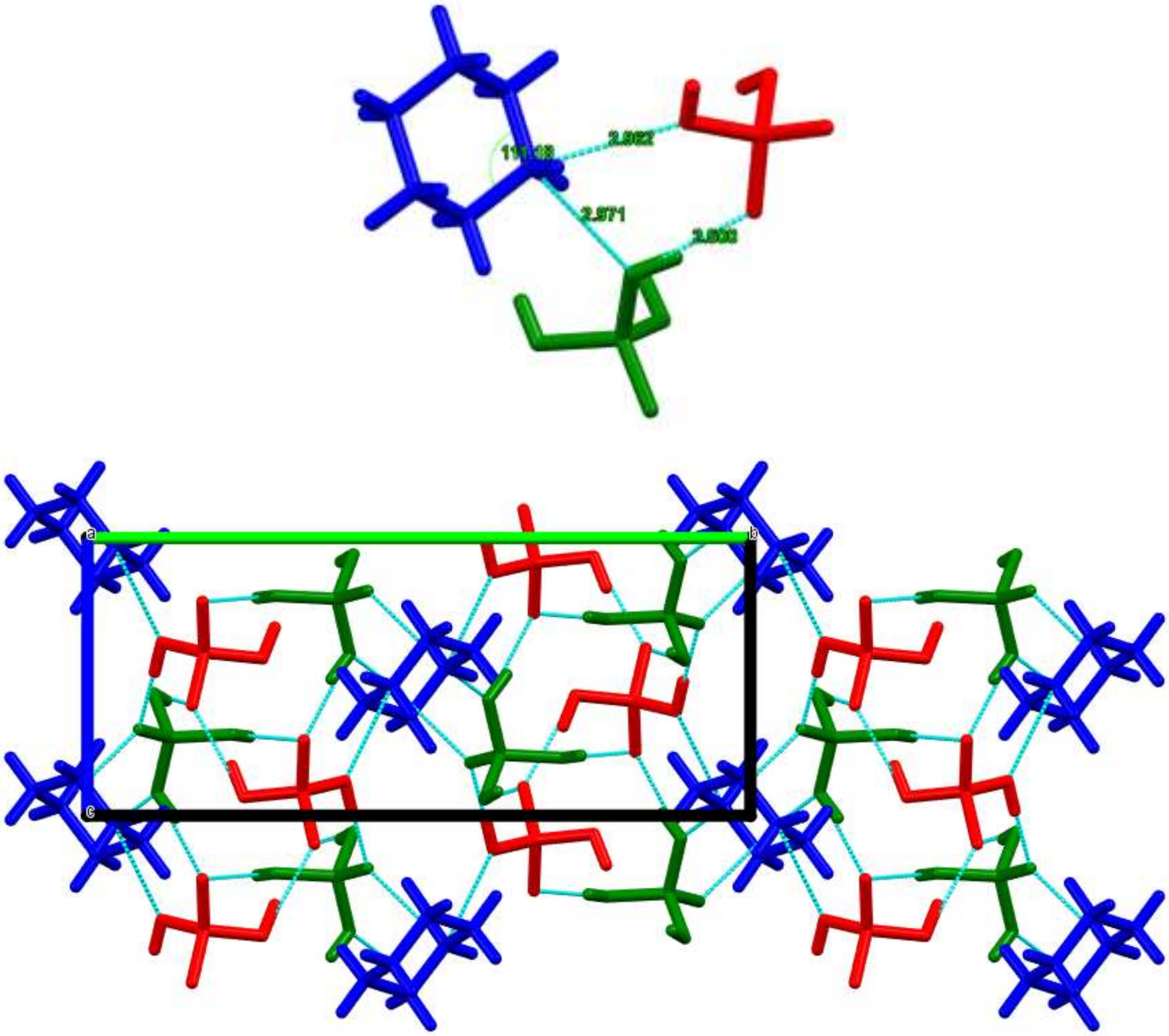

***Figure S1.6**. (Top) Structure of PIPDPP, bond lengths in Å and angle in degrees (°) (bottom) packing of PIPDPP along a-axis.*

1.7. AMQDPP

(a)

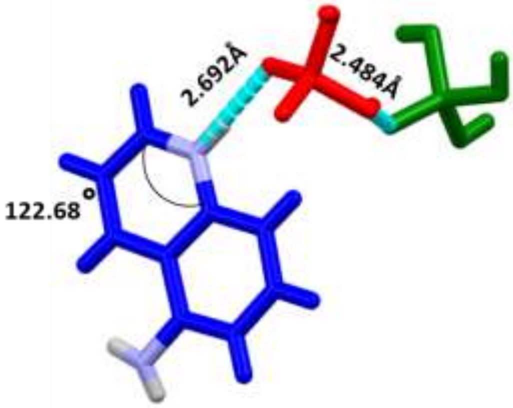

(b)

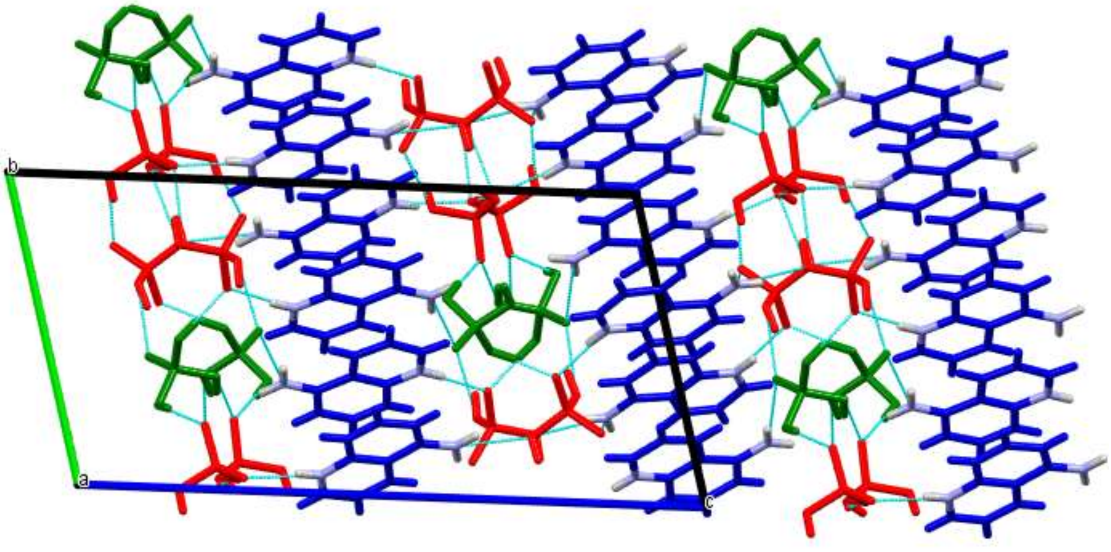

(c)

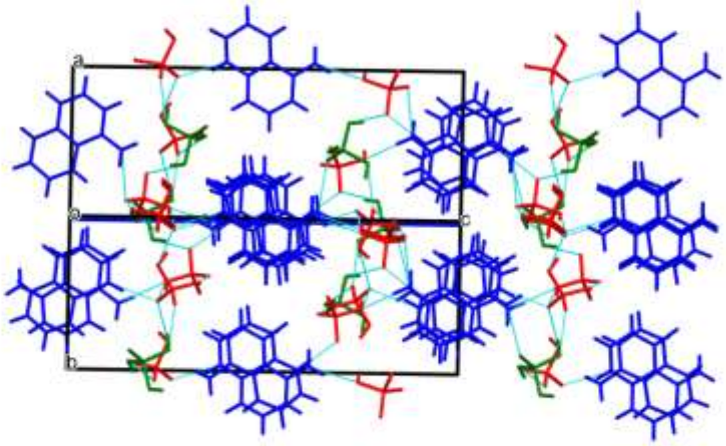

(d)

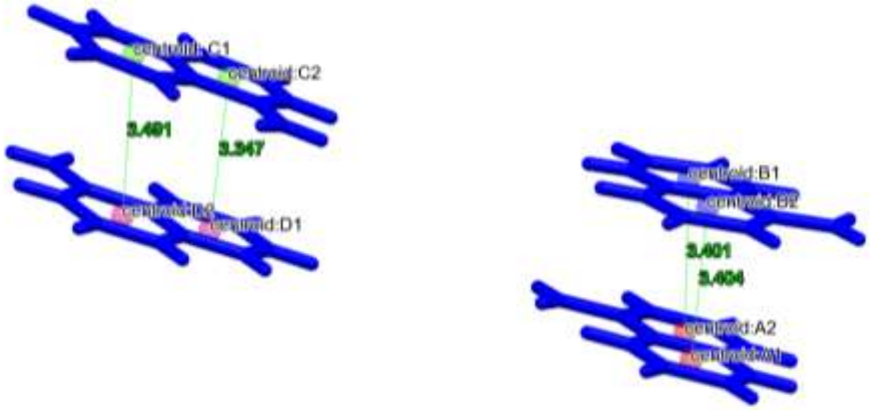

***Figure S1.7**. (a) Structure of AMQDPP, bond lengths in Å and angle in degrees (°) (b) packing of AMQDPP along b-axis (c) packing of AMQDPP along ab-axis (d) π-π stacking between stacked AMQ in Å*

1.8. DABDPP

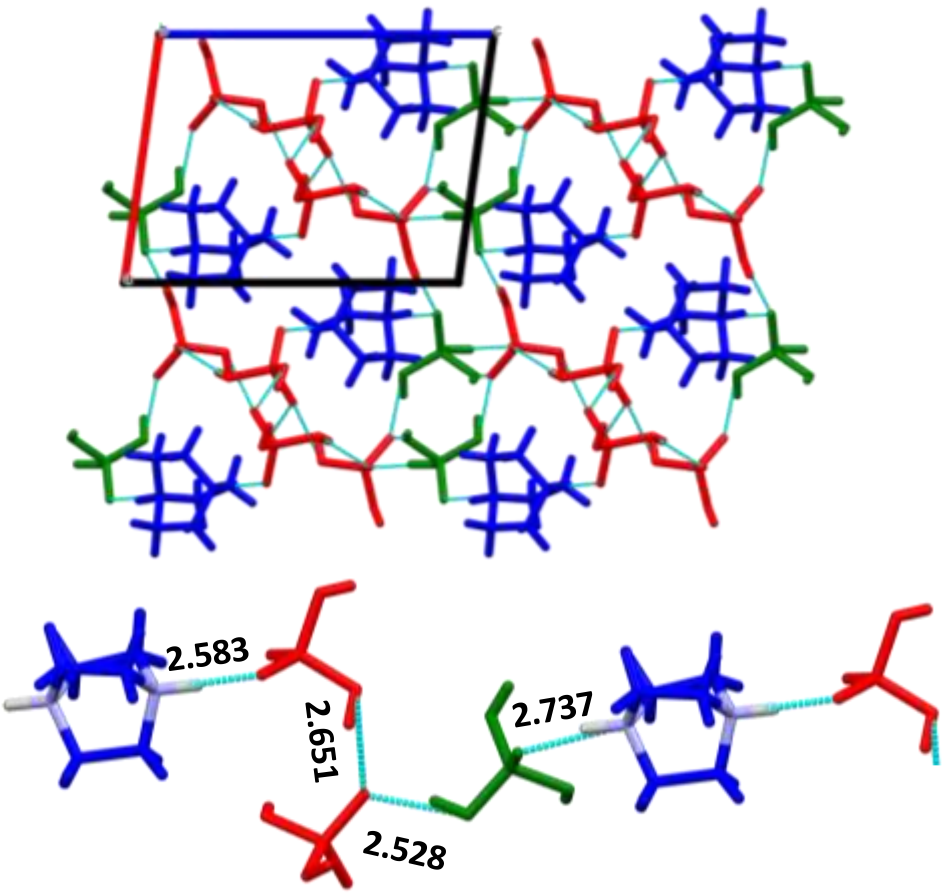

Figure S1.8. *(Top) Structure of DABDPP, bond lengths in Å and angle in degrees (°) (bottom) packing of DABDPP along b-axis*

1.9. ISQDPP

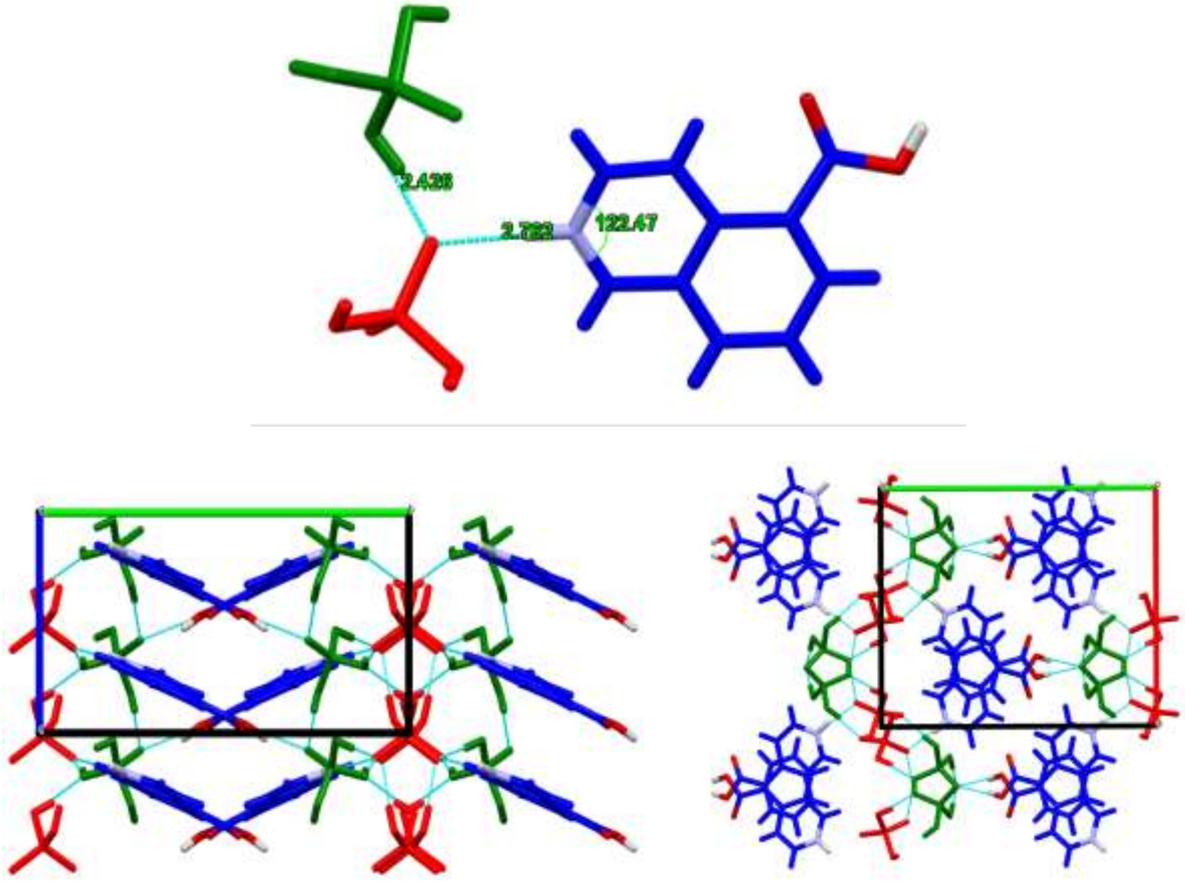

Figure S1.9. *(Top) Structure of ISQDPP, bond lengths in Å and angle in degrees (°) (bottom left to*

right) packing of ISQDPP along a-axis and c-axis.

1.10. LAMDPP

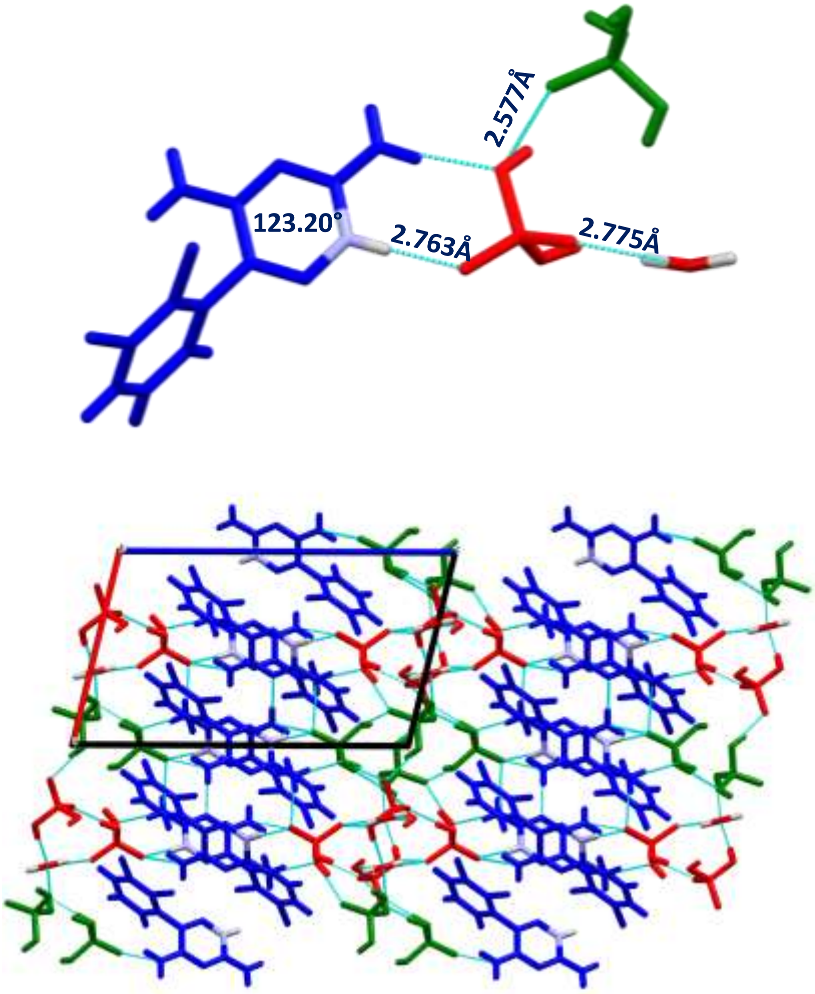

Figure S1.10. *(Top) Structure of LAMDPP, bond lengths in Å and angle in degrees (°) (bottom left to right) packing of LAMDPP along b-axis.*

Section S2: CSD Survey

Section 2.1. Table of DHP ICCs that do not contain PA as the neutral component found on the CSD

**ALORUQ**	**ASOQUC**	BOLKUJ	BORTAD
**CEBRAD**	**CITYOU**	**COGMIV**	**COWBOI**
**COWCUP**	**DASNUH**	**DAZJUL**	**DOXSUF**
**EYUTEZ**	**FACWOX**	**FAGXUI**	**HEXRIM**
**HICWOH**	**IKUSOY**	**IVUJOA**	**JUSMER**
**JUSMOB**	**JUSMUH**	KUZMUO	LETLUT
**LETMII**	**LETMEE**	LEZYOF	MIFRIC
MINVIP01	**NAHREV**	**NATNUU**	**NEHFOY**
**NIJXUC**	PEGXEH	POGGAT	**QIFPIG**
**QIFPOM**	**QIFPUS**	**QOTDOV**	**RERZOG**
**RERZUM**	RUZLOQ	**SAJZUA**	**SOBLED**
**SOBLIM**	**TISSOE**	**TITTIC**	**TUVDEU**
**TUVDIY**	**TUVDOE**	**VACSAW**	**VACSEA**
**VIDSOS**	**WAZWAX**	**WEDZUC**	**WEFBIU**
**XADBAK**	**YICHUQ**	**YIPVEB**	**YUBKIR**
**YUPVOV**	ENISIG	**ZIPDAG**	**ZINBAC**

Table S2.1. *List of cocrystals found on CSD, the bold REFCODES refer to the papers that contain DHP and tetrabutylammonium in their structure.*

Section 2.2. Table of DHP:PA cocrystals

**ADEZIV**	HISTPA10	PUJTAQ	WAVHOQ
APAFAA	JADZET	QALCAJ	WETDIJ
ECEDEW*	JAYXUD	QAYCAV	WEVXAX
**ELEVIC***	LIRZES*	RAFDUZ	XAZKAL
**ELEVOI**	LUKMEL*	RAVJUU*	XOZHEB*
ESAMAO	NIRFEB	SANDUH	XULJIZ
GEYVUE*	PAVTEN	**SOXFIV**	ZANFEC
GEYWUF*	PAZVAO01	TODNUV	ZEWZOT
HAKDED	**PETTIS**	TUPDEO	IPIPED*
HEGDED	PIKDES*	VEGKIB	TEBMAQ
DAZYAE	AGUAHP02	XUGFAH*	YEMJIJ
WOXKIG*	ZADPON	DAVCEK	**CURJAD**
IZOWID	JUKXUL	KIJHUJ	SIPRIT
OQEFAU	OQEFEY	HAGLAG	

Table S2.2*. List of cocrystals found on CSD, the bold REFCODES refer to the papers that report their structure as a cocrystal. Asterisks (*) indicates the structure is hydrated. Underlined structures were excluded from hydrogen bond motif analysis.*

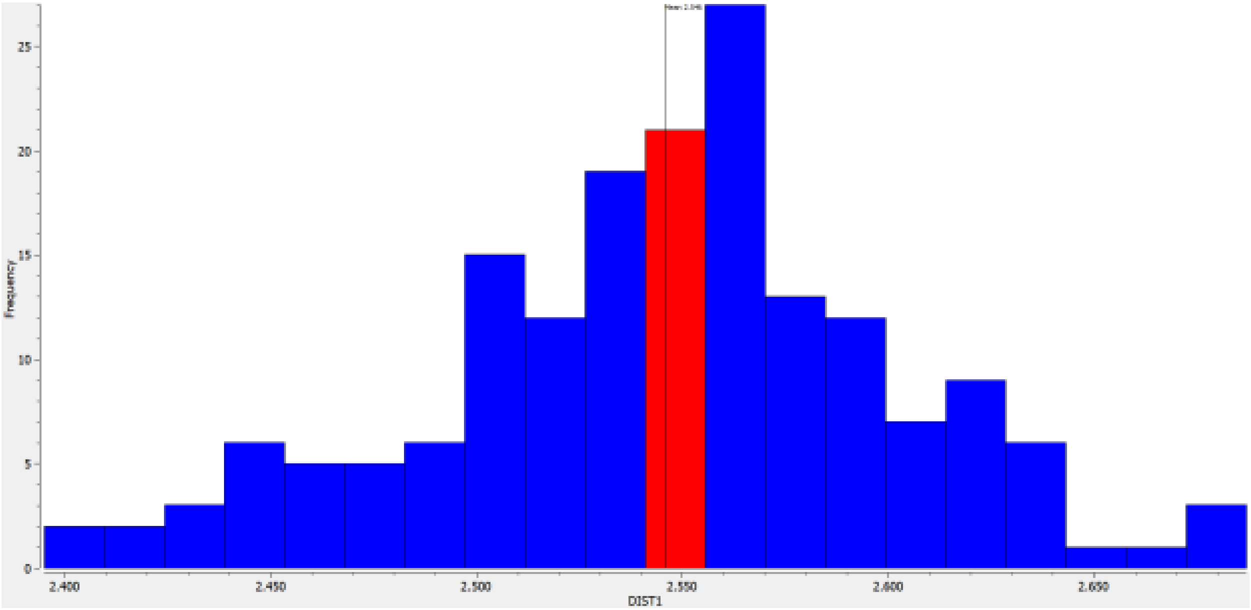

Fig S2.1*. Histogram of O-O distance between PO and POH, the mean bond distance is 2.546 Å.*

Section 2.3. Bond lengths of P-O and P=O (Å)

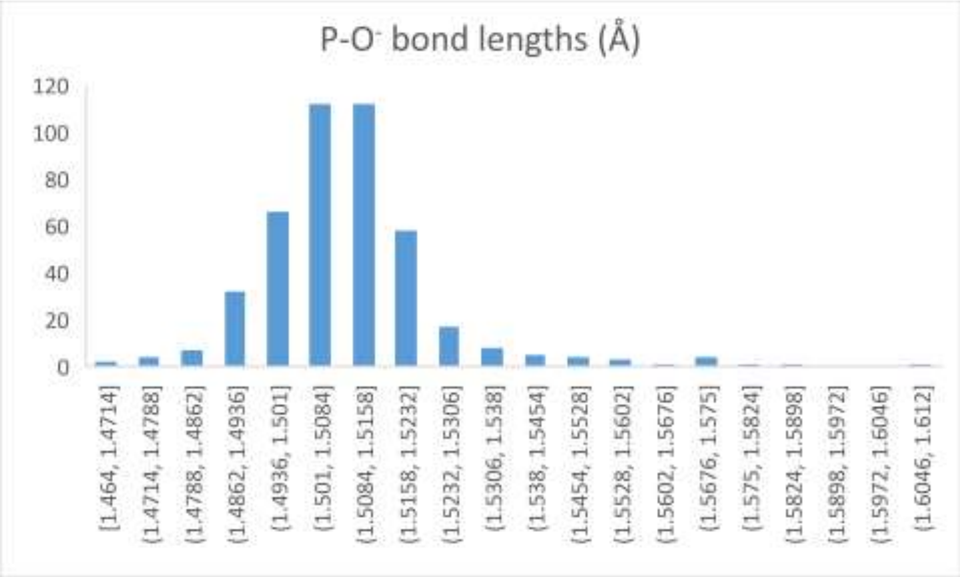

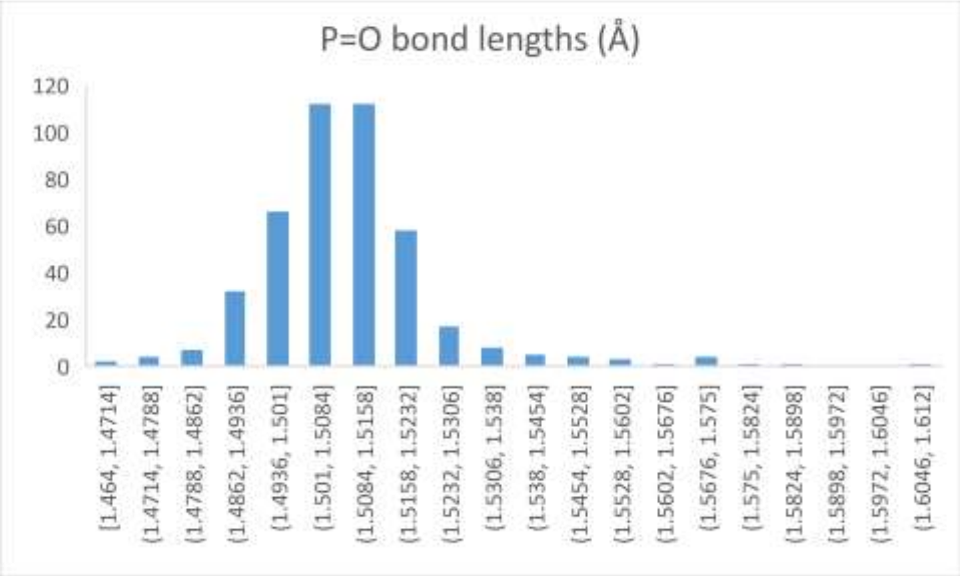

Histograms S2.2. *(Top) P-O$^-$ bond lengths and (bottom) P=O bond lengths from the CSD.*

3D coordinates; R-Factor <5%; single crystal structures only; organics only.

	P=O	P-O
Mean	1.5056	1.5095
SD	0.01396	0.01595
Mean±SD	1.4917-1.5196	1.4936-1.5254

So as expected, P=O is shorter than P-O$^-$ but cannot be differentiated from each other statistically.

Section 2.4. (O···O) bond distance between DPA and PA (Å)

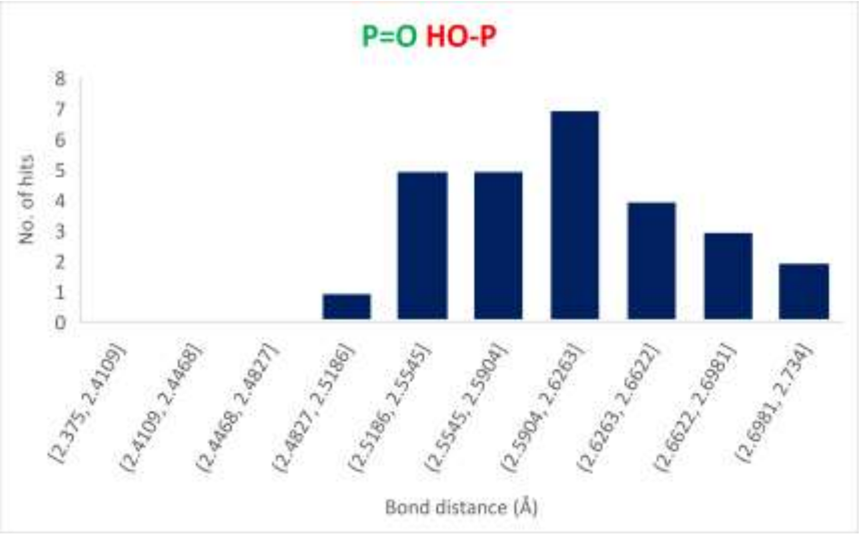

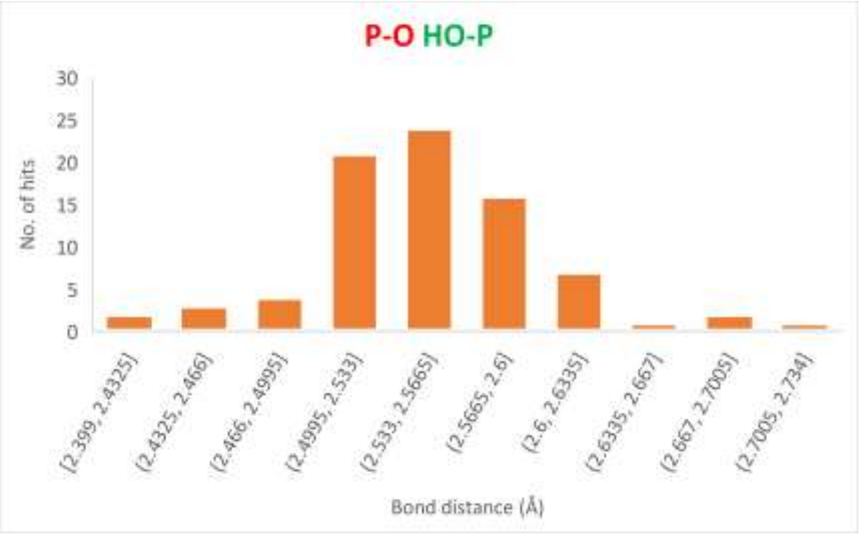

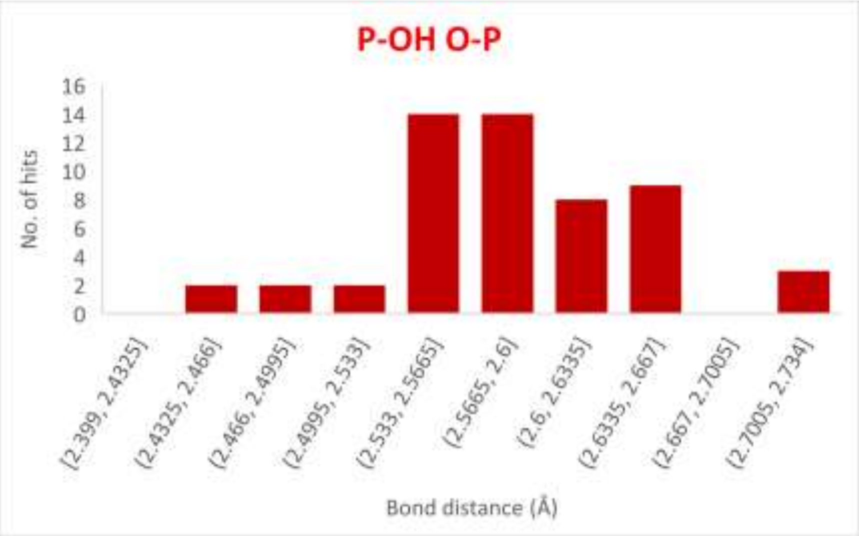

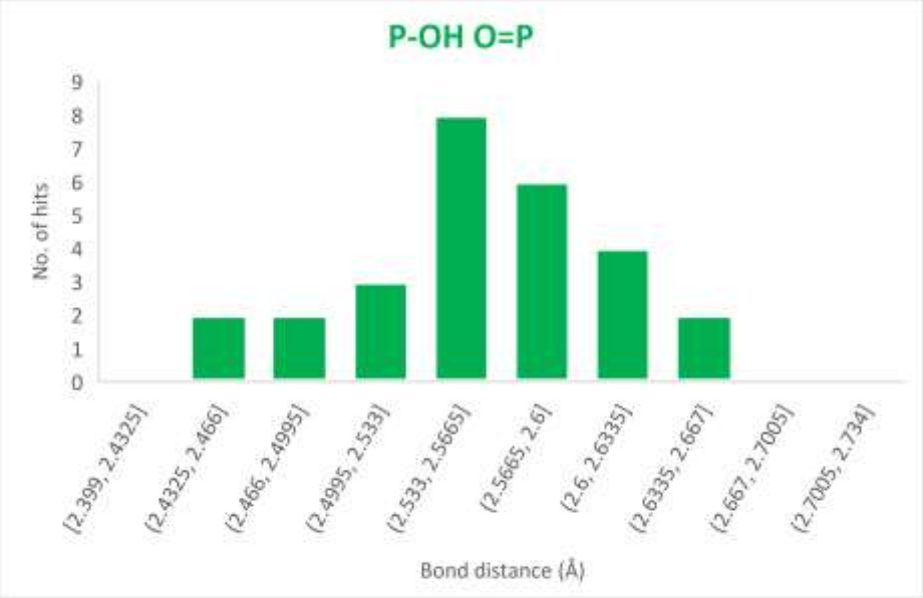

Histogram S2.4. *The four histograms above illustrate the average (O···O, Å) between the DHP and PA.*

	P-O HO-P	P=O HO-P	P-OH O-P	P-OH O=P
Mean (Å)	2.549	2.589	2.582	2.599
SD	0.048	0.047	0.052	0.052
Mean±SD (Å)	2.501-2.597	2.543-2.636	2.531-2.634	2.507-2.611

Analysis was carried out on the (O···O) between DHP and PA to investigate if there was a correlation between hydrogen bonds, with the hypothesis that the DHP-DPA interactions would be stronger and could help identify between DHP and PA. The results reveal there is no significant difference in bond distance between the molecules. There is slight increase in bond distance between PA (P=O) and DHP (P-OH) but this can be due to the neutral double in the PA, compared to the delocalized P-O found in DPA. According to the classification of Jeffrey, these are considered moderately strong hydrogen bonds.[1]

1. (a) Jeffrey, G. A.; Jeffrey, G. A., *An introduction to hydrogen bonding*. Oxford university press New York: 1997; Vol. 12. (b) Steiner, T., The hydrogen bond in the solid state. *Angewandte Chemie-International Edition* **2002,** *41* (1), 48-76.

Section 2.5. ΔpKa Rule

Cocrystal	Base			Acid (H_3PO_4)	ΔpKa
	pKa			pKa	
1	BPY[a]	pKa_1	3.17	2.15	1.02
		pKa_2	4.82	2.15	2.67
2	BPE[d]	pKa_1	3.17	2.15	1.02
		pKa_2	4.82	2.15	2.67
3	BPX[d]	pKa_1	3.17	2.15	1.02
		pKa_2	4.82	2.15	2.67
4	BPG[d]	pKa_1	3.17	2.15	1.02
		pKa_2	4.82	2.15	2.67
5	AZO[d]	pKa_1	3.17	2.15	1.02
		pKa_2	4.82	2.15	2.67
6	DAB[b]	pKa_1	2.97	2.15	0.82
		pKa_2	8.82	2.15	6.67
7	PIP[a]	pKa_1	5.68	2.15	3.35
		pKa_2	9.82	2.15	7.67
8	AMQ[a]	pKa_1	5.59	2.15	3.44
9	ISQ[a]	pKa_1	5.14	2.15	2.99
10	LAM[3]	pKa_1	5.7	2.15	3.55

Table S2.4. *ΔpKa values of cationic organic molecules.*

(a) Williams, R.; Jencks, W. P.; Westheimer, F. H. pKa Data Compiled by R. Williams. *University of Wisconsin-Madison, https://www.chem.wisc.edu/areas/reich/pkatable/pKa_compilation-1-Williams. pdf (Accessed 09 March 2022).*

(b) Ripin, D. H.; Evans, D. A. PKa's of Inorganic and Oxo-Acids. *The Evans* **2005**.

(3) O'Neil, M. *J.Merck Index*, 14th ed.; Merck Research Laboratories:Whitehouse Station, NJ, 2006.

(d) pKa values were given respective of 4,4'-bipyridine as pKa_2 values could not be obtained.

Section S3: Hydrogen bond motifs (HBMs)

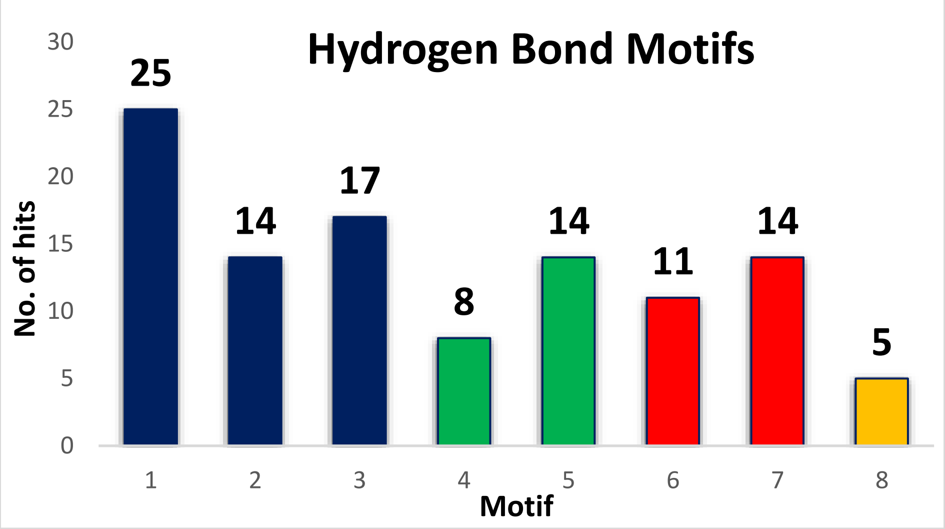

Chart S3.1. *Number of CSD structures with the motifs detailed in Figure 1: blue = dimer; green= trimer; red = tetramer; yellow is the next most common motif.*

	Dimer	Trimer	Tetramer	Pentamer	Hexamer	Heptamer	Octamer
1	AA	ANN	AANN	AAANN	AANAAN	AWAW WAN	WNAN WNAN
2	NN	AAN	ANAN	ANANN	AWNAWN	ANNAANN	AANN AANN*
3	AN	AAW	AAAW	AAANW	AAANAN	AAAANAN*	ANNA NNNN
4		AAA	ANNN	ANANW	AAAAAN	AAAAANN	
5		AWN	AWNW	AAAAN*	AAAANN		
6			NWNW	AWNNN*	AWNNAN		
7			AAWW		AAAAAA		
8			NNNN		NNNNNN		
9			AAAN		AAANNN		
10			ANWN		ANNANN		
11			AANW*		AANWAN*		
12			AWNN*		AANNAN		
13					WWANAA		

Table S3.1. Distinct hydrogen bond motifs compiled from 10 novel cocrystals and structures archived in the CSD. **A** indicates anionic dihydrogen phosphate (DHP), **N** indicates neutral phosphoric acid (PA) and **W** indicates a water molecule. * indicates motifs identified within the newly synthesised cocrystals.
